# Differentiating Aβ40 and Aβ42 in amyloid plaques with a small molecule fluorescence probe†

**DOI:** 10.1039/d0sc02060e

**Published:** 2020-05-11

**Authors:** Jing Yang, Biyue Zhu, Wei Yin, Zhihao Han, Chao Zheng, Peng Wang, Chongzhao Ran

**Affiliations:** Athinoula A. Martinos Center for Biomedical Imaging, Department of Radiology, Massachusetts General Hospital/Harvard Medical School Room 2301, Building 149, Charlestown Boston Massachusetts 021291 USA wangpeng@cpu.edu.cn cran@nmr.mgh.harvard.edu; School of Engineering, China Pharmaceutical University Nanjing 210009 China

## Abstract

Differentiating amyloid beta (Aβ) subspecies Aβ40 and Aβ42 has long been considered an impossible mission with small-molecule probes. In this report, based on recently published structures of Aβ fibrils, we designed iminocoumarin–thiazole (ICT) fluorescence probes to differentiate Aβ40 and Aβ42, among which Aβ42 has much higher neurotoxicity. We demonstrated that **ICTAD-1** robustly responds to Aβ fibrils, evidenced by turn-on fluorescence intensity and red-shifting of emission peaks. Remarkably, **ICTAD-1** showed different spectra towards Aβ40 and Aβ42 fibrils. *In vitro* results demonstrated that **ICTAD-1** could be used to differentiate Aβ40/42 in solutions. Moreover, our data revealed that **ICTAD-1** could be used to separate Aβ40/42 components in plaques of AD mouse brain slides. In addition, two-photon imaging suggested that **ICTAD-1** was able to cross the BBB and label plaques *in vivo*. Interestingly, we observed that **ICTAD-1** was specific toward plaques, but not cerebral amyloid angiopathy (CAA) on brain blood vessels. Given Aβ40 and Aβ42 species have significant differences of neurotoxicity, we believe that **ICTAD-1** can be used as an important tool for basic studies and has the potential to provide a better diagnosis in the future.

## Abstract 2

A small molecule fluorescence probe **ICTAD-1** was rationally designed for differentiating Aβ40 and Aβ42 in solutions and in Aβ plaques.

## Graphical abstract

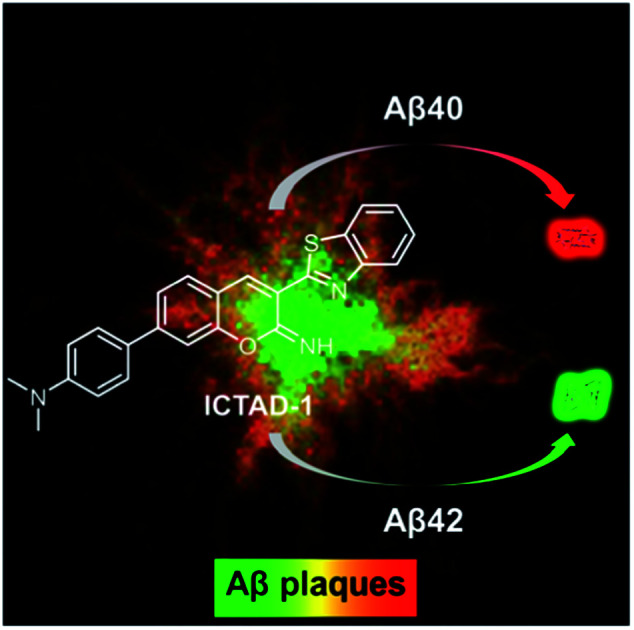

## Introduction

Amyloid beta (Aβ) plaques, one of the characteristic biomarkers for Alzheimer's disease (AD), have been discovered for more than 100 years in the postmortem brains of AD patients.^1,2^ However, the role of Aβ plaques in the pathology of AD has still been heavily debated, because the correlation between plaque burdens (numbers and areas) and the severity of AD is poor.^3–5^ In the plaques, Aβ40 and Aβ42 peptides are major constituents. Nonetheless, unlike the role of plaque, there is nearly no argument that Aβ42 has much higher neurotoxicity than Aβ40 does.^1,2,4,6,7^ Conceivably, differentiating Aβ40 and Aβ42 can considerably clarify the role of plaque in AD pathology. Unfortunately, small-molecule probes with such capacity are scarce.^7^

Due to the small difference in the amino acid sequence of the peptides, discovering small-molecule probes capable of differentiating Aβ40 and Aβ42 has been considered as an impossible mission. In our previous studies, inspired by the binding principles of antibodies for soluble and insoluble Aβs, we designed a series of small fluorescent molecules to selectively detect soluble Aβs,^8^ the likely biomarker for the early stage of AD pathology. Encouraged by the antibody's capability to specifically recognize Aβ40 or Aβ42 peptides, we hypothesized that it could be possible to differentiate Aβ40 and Aβ42 with a small molecule probe. Our design strategy is different from most previous studies, which are focused on adjusting optical properties to turn on (off) signals or make larger stokes shifts.^9–13^ Unfortunately, few probes have been designed based on the insights from the Aβ structures. In this report, we demonstrated that our designed small-molecule fluorescence probe, **ICTAD-1**, has the capacity to spectrally differentiate Aβ40 and Aβ42 *in vitro* and in the biologically relevant environment. We believe that our strategy could provide a new path for designing Aβ probes.

## Results

### Design of fluorescent imaging probes

It is obvious that the C-terminal of Aβ peptide is the key for designing small-molecule probes to distinguish Aβ40 and Aβ42. However, this is extremely challenging, due to the difference in only two amino acids (isoleucine–alanine). Nonetheless, it has been routinely performed in numerous laboratories with *anti*-Aβ42 antibodies to determine the contents of Aβ42 in cell media and brain tissues.^14–17^ These *anti*-Aβ42 antibodies were designed based on the epitope of the C-terminal of the peptide. This fact has bolstered us to believe that the properties of the C-terminal can be relied on to design our small molecule probes. In the past, X-ray structures of full-length Aβs were rare.^18–20^ However, in recent years, several detailed structures of Aβ fibrils have been published. Particularly, the advanced cryoEM technology has impressively facilitated Aβ structure studies.^21–23^

**Fig. 1:**
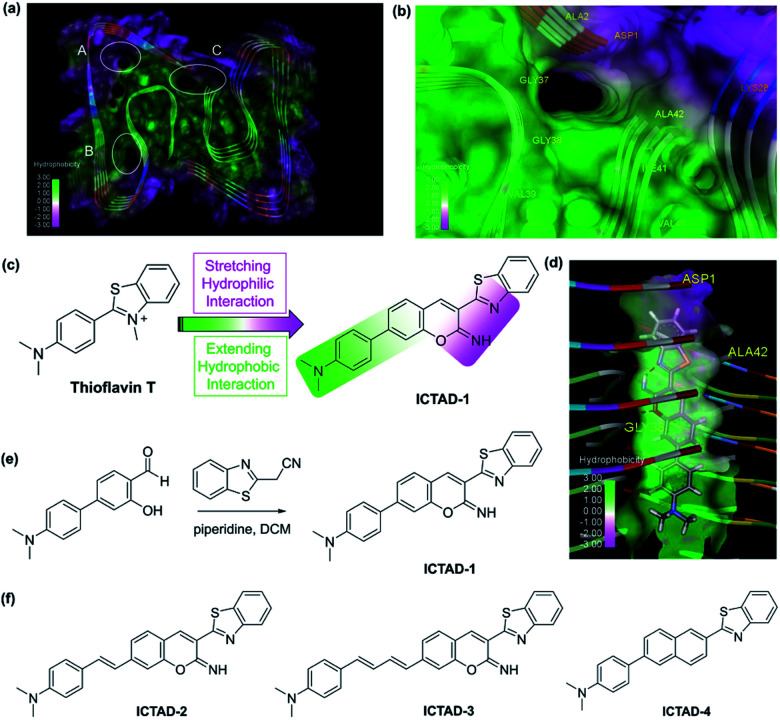
(a) The possible binding sites of Aβ42 protein (PDB: 5OQV); (b) binding site C of Aβ42 (PDB: 5OQV); (c) the design strategy of probe **ICTAD-1**; (d) the binding model of probe **ICTAD-1** with Aβ42 protein (PDB: 5OQV), the red dashed lines note hydrogen bonds; (e) the synthetic route for probe **ICTAD-1**; (f) the structures of compounds **ICTAD-2**, **ICTAD-3**, and **ICTAD-4**.

After having carefully surveyed the published structures of Aβ fibrils, we found several potential binding sites for designing probes for Aβ fibrils. The sites are A site, which locates in the hydrophilic N-terminal portion, B site, the hydrophobic mid-region, and C site, which is formed by a hydrophobic C-terminal from one Aβ peptide and a hydrophilic N-terminal from another Aβ peptide (Fig. 1a). Numerous small molecules have been reported as ligands of Aβ40 and Aβ42 species, including several sub-species such as soluble oligomers and insoluble fibrils. However, nearly all of the compounds have been designed to interact with the B site, which is the middle region of Aβ peptides.^8,10,24–35^ Obviously, this is not the suitable region for designing probes to differentiate Aβ40 and Aβ42. Surprisingly, to the best of our knowledge, no compound has been intentionally designed or validated to target the C-terminal and the C site. Interestingly, we noticed that, for the site C in most cases, multiple pieces of the Aβ peptide forms a gulf, in which one side consists of hydrophilic amino acids (K28, D1), and another side is hydrophobic of V39, V40, I41, and A42 (isoleucine–alanine), the last two amino acids in Aβ42 (Fig. 1b and S1†).

This interesting feature of the C site requires its binding ligands to meet the following criteria: (1) although Aβ40 and Aβ42 only have two amino acids difference, their hydrophobicity is very distinct. It is well known that, compared to Aβ40, Aβ42 is much stickier and considerably easier to aggregate. This is the key feature for designing probes to distinguish them, and this key feature requires the designed probes contain a hydrophobic moiety to interact differently with the hydrophobic side of the C-site; (2) in addition to the hydrophobic moiety, the ligand needs to contain a hydrophilic moiety to interact with the polar side; (3) the designed probe has a different binding affinity to Aβ40 and Aβ42 fibrils; and (4) the probes should have distinct fluorescence spectra and/or binding strength in the presence of Aβ40 and Aβ42 fibrils.

In terms of the fluorescent properties of small-molecule fluorescent probes, numerous compounds showed blue-shifting (hypsochromic shifting) after interacting with Aβs, suggesting they bind to a hydrophobic environment. However, benzothiazole analogues are distinct because they showed fluorescence red-shifting (bathochromic shifting),^36^ indicating this moiety can bind to a polar environment. The most typical compound is thioflavin T, which has been used as a gold standard for Aβ plaque staining, and its derivative PIB has been widely used in the clinic as a PET tracer for imaging Aβ deposits.^37–43^ Thioflavin T provides the classic emission of spectral red-shifting. However, thioflavin T cannot spectrally differentiate the Aβ fibrils, likely due to its incapacity to differently interact with the hydrophobic moiety of the C-site. Nonetheless, we consider thioflavin T is a good starting point for probe designing.

Based on the above consideration, the designed probe should be tilted between the hydrophilic and hydrophobic moieties with a vertical arrangement (parallel to the fibril axis) (Fig. 1d), while thioflavin T is too short to touch the hydrophobic moiety and this is why it does not have the differentiating capacity. Conceivably, extending the length of the hydrophobic patch of thioflavin T has the potential to achieve this goal (Fig. 1c). In this regard, we designed **ICTAD-1** (imino-coumarin–thiazole for AD), in which *N*,*N*-dimethyl-phenyl is for touching the hydrophobic moiety and for adjusting the binding affinity. In addition, iminocoumarin was introduced to stretch the hydrophilic moiety, which can enhance the binding through increasing hydrogen bonds (Fig. 1c). Moreover, the NH group in iminocoumarin could be served as a hydrogen donor to form an intramolecular hydrogen bond with benzothiazole moiety, leading to the enhanced planarity of the two heterocycles, which was distinctly different from the previously reported coumarin derivatives.

To validate this design strategy, molecular docking between **ICTAD-1** and Aβs was performed. The molecular docking results **ICTAD-1** with Aβ42 (PDB: 5OQV) showed that, indeed, the hydrophilic patch of C-site interacted with the stretched hydrophilic moiety of **ICTAD-1**, and the extended aromatic ring moiety interacted with the hydrophobic patch (Fig. 1d). The hydrophobic interaction between **ICTAD-1** with residues ALA2 and ILE41 of Aβ42 was observed. The oxygen atom in iminocoumarin formed an intermolecular hydrogen bonding with the NH of GLY38. Moreover, the intramolecular hydrogen bond was also observed. The docking score for **ICTAD-1** with Aβ42 at site C was −8.8476, which was lower than thioflavin T (−6.8242), indicating **ICTAD-1** might have better binding affinity to Aβ42 than thioflavin T. Similar docking results of **ICTAD-1** with other Aβ42 fibrillar structures (PDB: 5KK3 and 2NAO) were achieved (Fig. S2a and b†). We also carried out the docking studies of **ICTAD-1** with Aβ40 fibrillar structures (PDB: 2MVX). The docking results showed **ICTAD-1** could bind to the C site of Aβ40 through three intramolecular hydrogen bonds with residues HIS6 and GLU11 (Fig. S2c†). And it was observed that there were hydrophobic interactions between **ICTAD-1** with residues PHE4 and VAL40. Moreover, the docking score of **ICTAD-1** with Aβ40 was −8.2130, which was slightly lower than Aβ42. Similarly, **ICTAD-1** could also dock into the C sites of other Aβ40 fibrils (PDB: 2MPZ and 6SHS) (Fig. S2d and e†). Considering the difference in the hydrophobic environment at sites C in Aβ40 and Aβ42, these results indicated that **ICTAD-1** had the potential to bind and discriminate Aβ40 and Aβ42 fibrils.

To investigate whether the iminocoumarin moiety is necessary for binding, we designed **ICTAD-2** by replacing it with a naphthalene ring. In addition, we designed **ICTAD-3** and **-4** to further extend the hydrophobic moiety to investigate whether the compounds can better match with the hydrophobic patch in the C-site. The synthesis of **ICTAD-1** is straightforward, and its route is shown in Fig. 1e. The structures and synthetic routes for **ICTAD-2**, **-3**, and **-4** are shown in Fig. 1f and ESI.†

### Properties of **ICTAD-1**

**Fig. 2:**
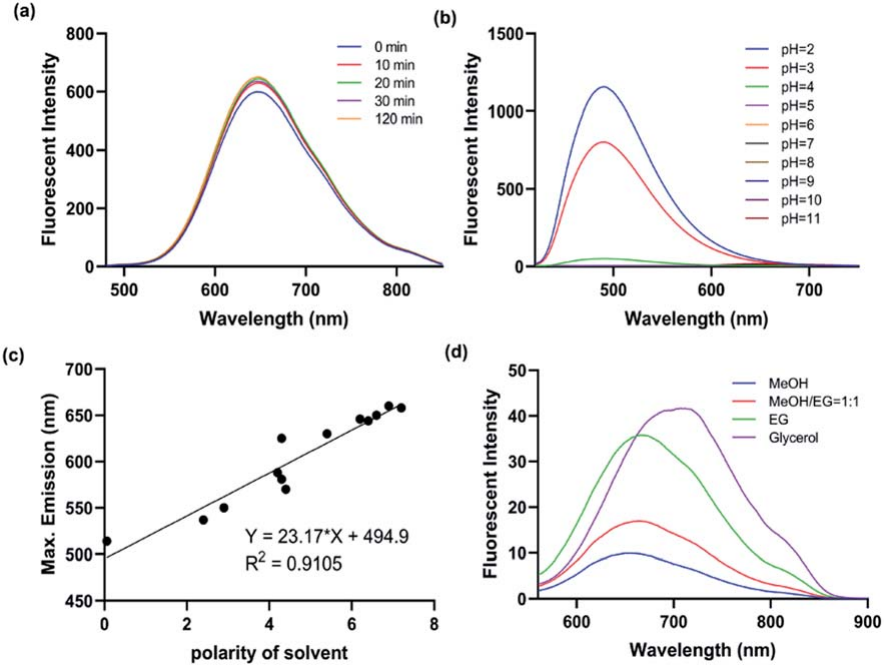
(a) The fluorescent spectra changes of **ICTAD-1** (10 μM) in DMF under the irradiation of daylight lamp for 120 min; (b) the fluorescent spectra of **ICTAD-1** (10 μM) in different pH aqueous solutions (1% DMF); (c) the linear relationship between the polarity of solvent and the maximum emission wavelength; (d) the fluorescence spectra of **ICTAD-1** (10 μM) in solvents of different viscosity, EG: ethylene glycol.

With the probe in hand, we firstly performed the photostability experiments in DMF to investigate whether **ICTAD-1** is stable under light (1500 joule per minute), and found that there were nearly no changes of fluorescence intensity after irradiating 120 minutes (Fig. 2a and S3a–d†), suggesting that **ICTAD-1** has excellent stability to resist photobleaching. To explore **ICTAD-1**'s responses towards different pH media, we titrated it within the range of pH 2–11, and found its absorbance intensity was consistent from pH 6–11. While we found that its fluorescence intensity decreased dramatically from pH 2–5, and its fluorescence is minimal under pH 7 (Fig. 2b and S4†), suggesting this probe has minimal fluorescence background signal and is suitable for *in vivo* imaging. With **ICTAD-1** in hand, we also investigate its absorbance and emission spectra in different solvents. We found that the absorbance is much less dependent (<30 nm changes) on solvent polarity, while its emission could be drastically changed in different solvents, and about 150 nm difference could be found from non-polar hexane to glycol. As we expected, **ICTAD-1** displayed much longer emission in polar solvents such as DMSO and glycol, indicating this probe can interact with a polar environment (Fig. 2c, d and S4†). Moreover, **ICTAD-1** exhibited higher quantum yield in the nonpolar solvents, such as hexane and toluene (Table S1†).

### Responses of **ICTAD-1** to Aβ fibrils in solutions

**Fig. 3:**
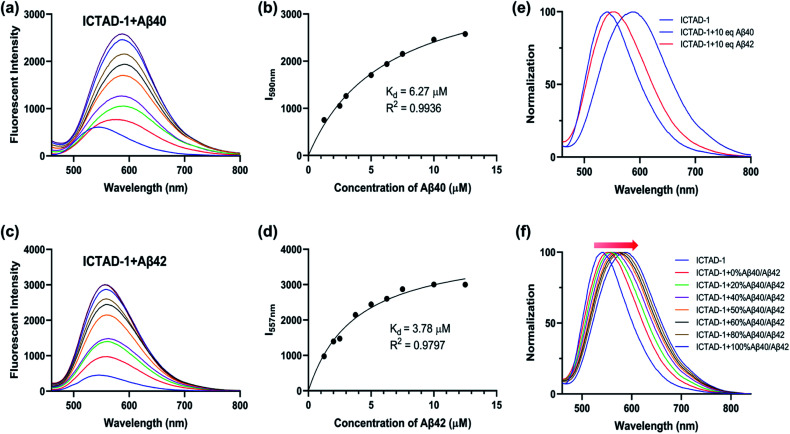
The fluorescent spectra of **ICTAD-1** (250 nM) in the absence and presence of Aβ40 (a) or Aβ42 (c); the binding curve of **ICTAD-1** (250 nM) with Aβ40 (b) or Aβ42 (d); (e) normalization fluorescent spectra of **ICTAD-1** (250 nM) with Aβ40 or Aβ42 (10 eq., 2.5 μM); (f) normalization fluorescent spectra of **ICTAD-1** (250 nM) with different percent of Aβ40 in Aβ42 (10 eq., 2.5 μM).

To examine **ICTAD-1**'s emission response to Aβ fibrils, we incubated 250 nM of the probe with different concentrations of Aβ40 and observed a consistent red-shifting of emission from 1.25 μM to 12.5 μM, suggesting the binding of **ICTAD-1** with Aβs is not 1 : 1 stoichiometry (Fig. 3a). Meanwhile, apparent fluorescence intensity increases could be observed with different concentrations of Aβ40. The largest shift was 47 nm and the largest intensity increase was over 6-fold. We also observed that the fluorescence intensity increased with time and it reached a plateau within 20 minutes (Fig. S5c and d†). The turn-on effect of **ICTAD-1** is likely due to twisted intramolecular charge transfer (TICT) upon binding to the fibrils, in which the planar configuration is preferred, and the rotation of the aromatic rings is restricted.^33,44^ From this study, we were also able to calculate a binding constant *K*_d_ = 6.27 μM for Aβ40, while *K*_d_ of Aβ42 was 3.78 μM, suggesting that the binding to Aβ40 fibrils was weaker than that for Aβ42 fibrils (Fig. 3a–d). In addition, we found that **ICTAD-1** provided excellent linear correlations with the concentrations of Aβ40 and Aβ42 fibrils in the range of 0–4 μM (Fig. S5†).

We then performed similar experiments to study the emission changes of **ICTAD-1** with Aβ42 fibrils and found consistent red-shifts. Remarkably, we observed that the degrees of emission peak shifting were different, and Aβ40 provided much larger red-shifting (Fig. 3e and f). This is expected because Aβ42 provided tight binding due to its higher hydrophobicity and crowed space, while Aβ40 is less crowded and provides loose binding. This is consistent with **ICTAD-1**'s binding constants for Aβ40/42. Interestingly, in the solutions of Aβ40/42 mixture, we found that the normalized spectral peaks shifted from shorter wavelengths (blue) to longer wavelengths (red) with the increasing ratio of Aβ40 component, and the shifted wavelength number was linear to the ratio of Aβ40/42 (Fig. S6†). In addition, we found that **ICTAD-1** had excellent selectivity over metal ions and other proteins, such as bovine serum albumin (BSA) and human serum albumin (HSA) (Fig. S7†).

To examine whether a naphthalene ring can be used to replace the iminocoumarin moiety in **ICTAD-1**, we performed similar solution tests with **ICTAD-2**, and found that **ICTAD-2** provided a slight red-shifting and a slight intensity decrease (Fig. S8a†), suggesting the hydrophilic iminocoumarin moiety is necessary for binding to the hydrophilic patch in Aβ fibrils. Different from **ICTAD-1**, **ICTAD-3** showed no apparent red-shifting, while it showed a slight intensity increase (Fig. S8b†). **ICTAD-4** showed an observable blue-shifting and a moderate intensity increase (Fig. S8c†). Since **ICTAD-2**, **-3**, and **-4** didn't have favorable properties for differentiating Aβ40/42, we didn't perform further investigation for these compounds.

### Differentiating Aβ40 and Aβ42 with **ICTAD-1***via* spectral unmixing imaging

**Fig. 4:**
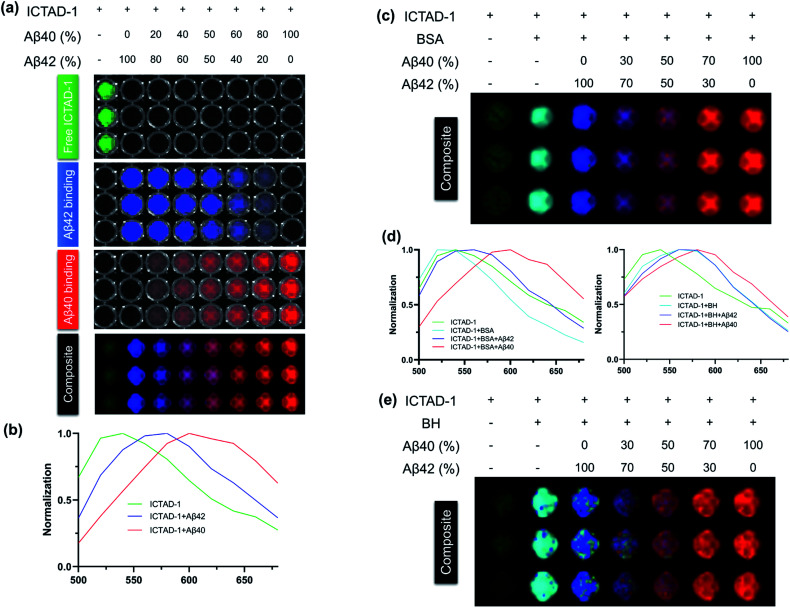
(a) Plate imaging of **ICTAD-1** (250 nM) in the presence of different ratios of Aβ40/Aβ42 (2.5 μM, 10 eq.) in 96-wells plate after unmixing. *E*_x_ = 430 nm, *E*_m_ = 500–680 nm. (b) Normalized unmixing spectra of **ICTAD-1** (250 nM) with or without Aβ40 and Aβ42; (c) plate imaging of **ICTAD-1** (250 nM) in the presence of different ratios of Aβ40/Aβ42 (2.5 μM, 10 eq.) in 96-wells plate after unmixing in the presence of BSA; (d) normalized unmixing spectra of **ICTAD-1** (250 nM) with or without Aβ40 and Aβ42 in the presence of BSA or mouse brain homogenate (BH); (e) plate imaging of **ICTAD-1** (250 nM) in the presence of different ratios of Aβ40/Aβ42 (2.5 μM, 10 eq.) in 96-wells plate after unmixing in the presence of mouse brain homogenate.

To explore whether **ICTAD-1** can be used to quantify the amount of Aβ40 and Aβ42 fibrils, spectral unmixing with mixtures of these fibrils was performed (Fig. S9†). First, we used pure Aβ40 and Aβ42 fibrils solutions to establish the spectra on a 96-well plate. The linear relationships could be observed for both fibrils (Fig. S10†), suggesting that spectral unmixing is feasible to quantify the contribution of each component in the mixture. The detection limitations are 3.3 nM and 28.3 nM for Aβ40 fibrils and Aβ42 fibrils respectively. Then we conducted library-based spectral unmixing to separate the ratios of these two fibrils. Indeed, we found that it was feasible to deconvolute the signals with the characteristic spectra of free **ICTAD-1** (green), **ICTAD-1** with Aβ40 fibrils (red), and Aβ42 fibrils (blue) (Fig. 4a and b).

Next, to investigate whether spectral unmixing is feasible in a biologically relevant environment, we performed the above experiments in the presence of bovine serum albumin (BSA) and mouse brain homogenate (BH). As we expected, spectral unmixing could be used to deconvolute the signals from the free probe (green), binding with BSA or BH (cyan), binding with Aβ40 (red) and Aβ42 (blue) (Fig. 4c–e), suggesting it is possible to spectrally differentiate the Aβ fibrils in biologically relevant environments.

### Tissue staining and spectral unmixing with **ICTAD-1**

**Fig. 5:**
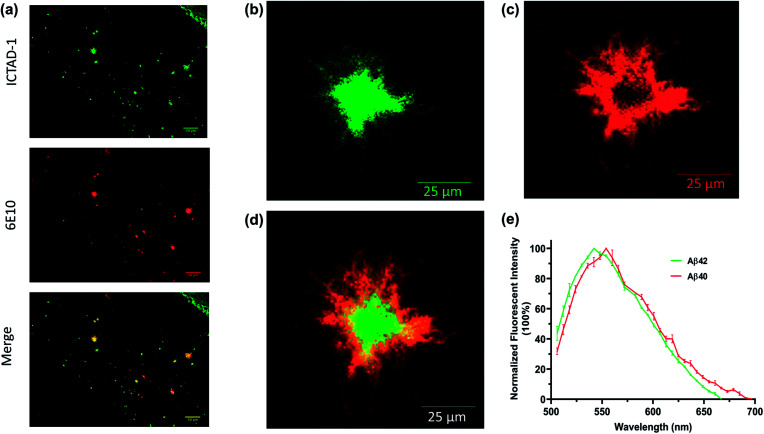
(a) The brain tissues from 5xFAD mice were co-stained with **ICTAD-1** and antibody 6E10; (b) the core of Aβ plaque stained with **ICTAD-1**; (c) the periphery of Aβ plaque stained with **ICTAD-1**; (d) merged image of (b) and (c); (e) the normalized unmixing fluorescent spectra of Aβ plaques stained with **ICTAD-1** (*n* = 3).

We first explored whether **ICTAD-1** could provide a high quality of plaque staining, and found that plaques on brain slides of 5xFAD mice can be sharply visualized after the slide was incubated with the probe for 15 minutes (Fig. 5a), and the labeled plaque showed excellent colocalization with 6E10 antibody staining (Fig. 5a), which is specific to Aβs. This result suggested that **ICTAD-1** was able to specifically label Aβ plaques.

The previous results inspired us to explore the capability of **ICTAD-1** in differentiating Aβ40 and Aβ42 fibrils in the plaques. Then we relied on confocal imaging that has been equipped with a spectral unmixing function. Interestingly, the spectra from the core most closely resembled the one for Aβ42 *in vitro* studies, whose peak wavelength was shorter. Meanwhile, the spectra from the periphery were similar to the one from Aβ40 fibrils *in vitro*, whose peak has a longer wavelength (Fig. 5b–e and S11†). The peak wavelength difference from the two spectra was 12 nm (Fig. 5e). Reportedly, Aβ42 is the dominant species in the core of plaques,^45–48^ which is consistent with our spectral unmixing results. Taken together, these results suggested that **ICTAD-1** could be used to quantify the contents of Aβ42/Aβ40 from brain slides. This also indicates that it is possible to quantify the subspecies in human brain tissue, which is very important to clarify the contributions of Aβ42 fibrils to the AD pathology.

### *In vivo* two-photon imaging with **ICTAD-1**

**Fig. 6:**
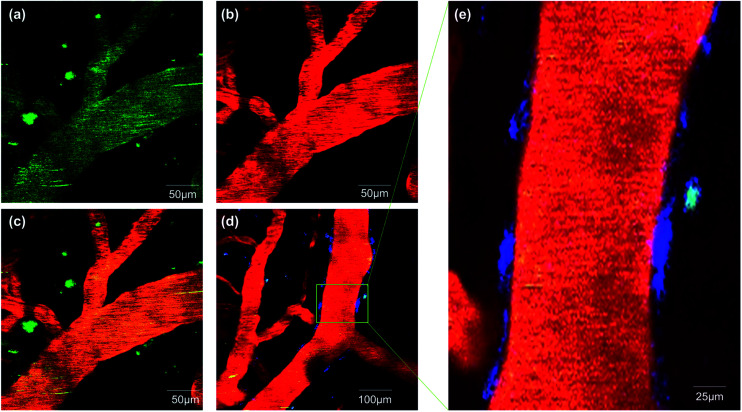
Two-photon fluorescent images in 5xFAD mice after tail intravenous injected with **ICTAD-1** and FITC–dextran. (a) The fluorescence from **ICTAD-1** in emission channel of 570–620 nm; (b) the fluorescence from FITC–dextran in emission channel of 500–550 nm; (c) the merged image of (a) and (b); (d) fluorescence imaging of Aβ plaques, CAA and blood vessel; (e) partial enlarged detail of (d).

To validate whether **ICTAD-1** can be used for *in vivo* imaging, we performed two-photon microscopic imaging with 15 month old 5xFAD mice. We first intravenously (iv) injected the mouse with FITC–dextran as the contrast agent for blood vessels. After 5 minutes, we iv injected **ICTAD-1** (1 mg kg^−1^), and imaged the mouse at 5 minutes post-injection of **ICTAD-1**. As expected, the plaques can be easily identified, due to the excellent contrast (Fig. 6a–c), suggesting that the probe can cross the brain–blood barrier (BBB) and can label plaques *in vivo*. Interestingly, we experienced some difficulties to identify cerebral amyloid angiopathy (CAA) on the vessels, which can be found from its autofluorescence (blue in Fig. 6d and e). These results indicated that **ICTAD-1** has weak capacities to label CAAs, in which Aβ40 is the dominant species.^49,50^ This result could be possibly explained with our *in vitro K*_d_ data, which indicated that **ICTAD-1** had much stronger binding to Aβ42, while it showed weaker binding to Aβ40. This *in vivo* data also suggested that differentiating Aβ40 and Aβ42 *in vivo via* different binding strength (*K*_d_) was feasible.

## Discussion

Aβ plaques have been discovered for more than 100 years, however, their roles in AD pathology are still poorly defined. The complicated dynamics and contributions of each component are the likely factors for the perplex. It is well documented that Aβ40 and Aβ42 are the major components of the plaques. Nonetheless, it is not clear what is the contribution weight of each Aβ subspecies to the pathology, and whether the content of Aβ42 in plaques has better correlations with the severity of the disease. With our probe, it is possible to partially solve the mysteries, and we may have the capacity to quantify how much Aβ42 is in each plaque, each brain slide, and specific brain areas. Conceivably, it is also possible to quantify Aβ42 contents for a whole-brain through serial slide imaging. With such a tool, we will be well equipped to answer those basic mechanism questions and will clarify the questions around postmortem plaques, which will provide more diagnostic information.

Thioflavin T reportedly showed slight difference of fluorescence lifetimes in pure solutions for Aβ40 and Aβ42 fibrils,^51^ however, it remains unknown whether the differences can be applied under physiological conditions. Kung *et al.*^52^ reported that radioligand [^125^I]DMTZ had certain preference for Aβ42-positive cerebral amyloid angiopathy (CAA). However, it is not clear whether it has the capacity to differentiate Aβ40/42 in the plaques.

In this report, we also demonstrated that **ICTAD-1** could be used to separate Aβ40/42 using spectral properties *in vitro* and binding strength *in vivo*. The spectral unmixing is easy to perform on a regular confocal microscope, suggesting our method can be easily adapted by other biological-oriented laboratories. From the slide spectral unmixing, the separation of emission peaks of Aβ40/42 was 12 nm, which may indicate that the separation of Aβ40/42 is imperfect. However, it is possible to achieve a cleaner separation (a larger difference in the emission peak) if a better algorithm is developed.

The difference of *K*_d_ for Aβ40/42 could be utilized for designing PET tracers that have much high specificity for Aβ42, the most toxic species. Our *in vivo* two-photon imaging indicated that **ICTAD-1** had certain binding preference towards Aβ42, and this could be considered as clues for designing for Aβ42 specific PET tracers. In addition, it is also tremendously important if we can differentiate CAA and plaques with a PET tracer, which is not currently available. Reportedly, Aβ40 is the major component of CAAs.^49,50^ Our data from this report suggests that our probe and its analogues may have the potential to differentiate plaques and CAAs. With such a probe, we will be able to dissect the vascular CAA contribution and plaque contribution to AD pathology.

Our studies also pointed to several possible directions for future AD research. First, we may have the capacity to monitor therapy that is prone to reduce Aβ42. Second, it is possible to design drugs that specifically target Aβ42. Lastly, we will be able to investigate whether the specific reduction of Aβ42 is a validated approach for future drug development.

## Conclusions

In summary, we demonstrated that **ICTAD-1** was able to spectrally differentiate Aβ40/42 in solutions, in plasma and in brain tissues. **ICTAD-1** has the potential to dissect the toxicity contributions of Aβ40 and Aβ42, and could be considered as a lead compound for developing Aβ42 specific PET tracers.

## Ethical statement

All animal experiments were performed in strict accordance with the NIH guidelines for the care and use of laboratory animals (NIH Publication No. 85-23 Rev. 1985) and was approved by the Institutional Animal Care and Use Committee at Massachusetts General Hospital.

## Conflicts of interest

There are no conflicts to declare.

## Supplementary Material

SC-011-D0SC02060E-s001
