# Supplementary material for: Differentiating Aβ40 and Aβ42 in amyloid plaques with a small molecule fluorescence probe
Source: Chem Sci. 2020 May 11;11(20):5238–45. doi: 10.1039/d0sc02060e (PMC8159352; doi:10.1039/d0sc02060e)
Supplement: SC-011-D0SC02060E-s001 [file SC-011-D0SC02060E-s001.pdf]

Electronic Supplementary Material (ESI) for Chemical Science.
This journal is © The Royal Society of Chemistry 2020

Supporting Information

Differentiating Aβ40 and Aβ42 in amyloid plaques with a small molecule fluorescence probe

Jing Yang, Biyue Zhu, Wei Yin, Zhihao Han, Chao Zheng, Peng Wang*, and Chongzhao Ran*

Table of Contents

Materials and instruments ... S3
Experimental procedures ... S3
Synthesis of probes ... S5
Table S1 ... S8
Figure S1 ... S8
Figure S2 ... S9
Figure S3 ... S9
Figure S4 ... S10
Figure S5 ... S10
Figure S6 ... S11
Figure S7 ... S11
Figure S8 ... S12
Figure S9 ... S12
Figure S10 ... S13
Figure S11 ... S13
Figure S12 ... S14
Figure S13 ... S14
Figure S14 ... S15
Figure S15 ... S15
Figure S16 ... S16
Figure S17 ... S16
Figure S18 ... S17
Figure S19 ... S17
Figure S20 ... S18

Figure S21 .... S18
Figure S22 .... S19
Figure S23 .... S19

Materials and instruments

All chemicals were purchased through commercial vendors and used without further purification. Absorption spectra were recorded on SpectraMax (Molecular Device, San Jose, CA). A fluorescence spectrophotometer F-7100 (Hitachi High-Tech Corporation, Japan) was employed fluorescent spectra recording. ^{13}C NMR and ^{1}H NMR spectra were collected on a JOEL 500-MHz Spectrometer (JEOL USA, Inc.), and the values of δ are in ppm respect to TMS. LC-MS was carried out on an Agilent 1200 Series apparatus with an LC/MSD trap (Agilent Technologies) and Daly conversion dynode detector with UV detection at 254 nm. Tissue imaging was performed on Nikon Eclipse 50i (Nikon Corporation). Two-photon imaging in vivo was conducted on an Olympus BX-51 microscope (Olympus Life Science). Transgenic female 5xFAD mice and age-matched wild-type female mice were purchased from Jackson Laboratory. All animal experiments were performed in strict accordance with the NIH guidelines for the care and use of laboratory animals (NIH Publication No. 85-23 Rev. 1985) and was approved by the Institutional Animal Care and Use Committee at Massachusetts General Hospital. An IVIS Spectrum animal imaging system (Perkin Elmer) was used for in vitro plate imaging.

Experimental Procedures

Spectra and quantum yield of ICTAD-1 in different organic solvents. The absorption spectrum of ICTAD-1 (10 μM) in the solution of PBS (10 mM, pH 7.4, 1% DMF) was recorded on SpectraMax Microplate Reader (Molecular Device, San Joe, CA). The ICTAD-1 stock solution was prepared in DMF and all testing solutions contained a final concentration of 1% DMF. The fluorescent spectra of ICTAD-1 (10 μM) in a solution of different organic solvents were performed on the F-7100 fluorescence spectrophotometer with Ex = 430 nm, slit 2.5/2.5, and 400 V for PMT. The quantum yields of ICTAD-1 were calculated based on Rhodamine B in ethanol (Φ_F = 0.64).

Spectroscopy of ICTAD-1 in solvents with increasing viscosity. Emission spectra of ICTAD-1 (10 μM) in solutions of methanol, methanol & ethylene glycol (1:1, v/v), ethylene glycol (EG), ethylene glycol & glycerol (1:1 v/v) and glycerol (Gly) at room temperature were performed on the F-7100 fluorescence spectrofluorometer.

The light stability of ICTAD-1. Light stability of ICTAD-1 (10 μM) in DMF was investigated under the irradiation of an incandescent lamp (25 W, 120 V, GE Lighting). The absorption and fluorescent spectra at different time points were recorded and the duration was 120 minutes.

pH effects on the spectroscopy of ICTAD-1. Absorption spectra of ICTAD-1 (10 μM) in different pH aqueous solutions (1% DMF) was recorded on SpectraMax Microplate Reader. The fluorescent spectra of ICTAD-1 (10 μM) in different pH aqueous solutions (1% DMF) were recorded on the F-7100 fluorescence spectrophotometer with Ex = 430 nm, slit 2.5/2.5, and 400 V for PMT.

Molecule docking studies. Molecular docking simulations were conducted with the Molecular Operating Environment (MOE) software version 2014.0901 (Chemical Computing Group, Montreal, Canada). The crystal structures of Aβ (PDB ID: 5OQV, 2MVX, 6SHS, and 2NAO) were obtained from the Protein Database (www. rcsb.org). Hydrogen atoms were added to the protein and energy minimization was performed using the MMFF94 force field. Docking of the ligands into the active site of the Aβ was performed by the triangle matcher docking protocol of the MOE program. As the refinement method, the force field was chosen. The resulting poses are then scored using the GBVI/WSA dG docking scoring function. The figures were generated using Discovery Studio 2019 Client.

Preparation of Aβ40 and Aβ42 aggregates/fibrils. Aβ40 (trifluoroacetic acid) peptide powder was dissolved in PBS (10 mM, pH 7.4, 25 μM). The solution was stirred at 37 ^{o}C for 3 days to give Aβ40 aggregates/fibrils. This stock solution was stored at 4 ^{o}C. Aβ42 aggregates/fibrils were prepared following the same procedure as Aβ40 fibrils.

Fluorescence responses of ICTAD-1 in the presence of Aβ species. Fluorescence spectra of ICTAD-1 (250 nM) in PBS (10 mM, pH 7.4, 1% DMF) were recorded before and after the addition of different concentrations of Aβs (0-12.5 μM) with Ex = 430 nm, slit 5/5, and 700 V for PMT. Binding constant values (Kd) were calculated with Prism.

Kinetic study of ICTAD-1 in the presence of Aβ species. ICTAD-1 (250 nM) was incubated with Aβ (2.5 μM, 10 eq) in PBS (10 mM, pH 7.4, 1% DMF), and fluorescent spectra were recorded at different time points within 20 min (Ex = 430 nm, slit 5/5, 700 V for PMT).

Selectivity of ICTAD-1 towards Aβ fibrils over metal ions, BSA and HSA. Fluorescent spectra of ICTAD-1 (10 μM) with different analytes in PBS (10 mM, pH 7.4, 1% DMF) were recorded, and concentrations of metal ions, amino acids, and thiols were 1 mM. PBS solutions of BSA and HSA were also tested with ICTAD-1 (10 μM) (Ex = 430 nm, slit 5/5, 400 V for PMT).

Plate imaging of ICTAD-1. ICTAD-1 (250 nM) was incubated with different concentrations of Aβ40 or Aβ42 (2.5 μM, 10 eq) in PBS (10 mM, pH 7.4, 1% DMF). The resulting solution was added to wells on a 96-well plate, and the final volume of each well was 200 μL. Triplicate samples were used for each condition. Plate images were recorded on the IVIS System. Moreover, the plate imaging of ICTAD-1 with Aβ40 fibrils and Aβ42 fibrils (2.5 μM, 10 eq) in the presence of BSA (2.5 μM, 10 eq) or mouse brain homogenate (BH, 0.1 mg/mL) was also performed similarly to the above procedure.

In vitro histological staining. A fresh brain tissue from a 24-month old APP/PS1 mouse was fixed in 4% formaldehyde for 24 hours and transferred into 30% sucrose at 4 °C until the tissue sunk. Then the tissue was embedded in OCT with gradual cooling over dry ice. The OCT embedded tissue block was sectioned into 25-μm slice with a cryostat. The tissue was washed in diluting buffer (0.4% Triton X-100, 1% goat serum,

2% BSA in TBS) for 3x10min, then blocked in 20% goat serum for 30min at room temperature. Then the slice was incubated with primary antibody 6E10 overnight at 4°C. After washed with diluting buffer for 3x10min, the slice was incubated with secondary antibody for 2 hours at room temperature. Then the slice was washed with TBS for 3x10min. 25 uM of ICTAD-1 in 20% ethanol/PBS was prepared as the staining solution. The brain slice was incubated with freshly prepared staining solution for 15 min at room temperature and then washed with 50% ethanol for 2x1min, followed by washing with double distilled water twice. Then the slice was covered with FluoroShield mounting medium (Abcam) and sealed with nail polish. Florescence images were obtained using the Nikon Eclipse 50i microscope with blue and red-light excitation channel.

Confocal spectral unmixing imaging. The above brain slice was placed under a Nikon confocal microscope (A1R HD25). Excitation laser of 468 nm was used, and emission was collected from 505 nm to 690 nm with 2.5 nm steps. A region of interest (ROI) was selected from the core and peripheral area of a plaque respectively, and a spectrum from each ROI was extracted and stored into the system as the spectral library. Spectral unmixing was performed with the spectra stored in the library. The unmixing spectra was achieved by averaging the data collected from three individual plaques (core and periphery respectively).

In vivo two-photon imaging. These animal experiments were performed in strict accordance with the NIH guidelines for the care and use of laboratory animals (NIH Publication No. 85-23 Rev. 1985) and was approved by the Institutional Animal Care and Use Committee at Massachusetts General Hospital. A 15-month-old 5xFAD female mouse was anesthetized with 2% isoflurane, and a cranial imaging window was surgically prepared as described [2]. Before ICTAD-1 injection, two-photon images of capillary were acquired using the 800-nm laser (Prairie Ultima) with 500 to 550 nm emission by injection of FITC-Dextran. A bolus i.v. injection of ICTAD-1 (1.0 mg/kg in a fresh solution of 15% cremophor, 15% DMSO, and 70% PBS) was administered. The images were acquired at 5 minutes after the ICTAD-1 injection and with emission channels of 500-550 nm and 570-620 nm. For imaging, we used a two-photon microscope (Olympus BX-51) equipped with a 20× water-immersion objective (0.45 numerical aperture; Olympus) [3]. Single Images and Z-series images were collected with a 512 × 512-pixel resolution. Image analysis was performed with ImageJ software.

Synthesis of probes

Synthesis of ICTAD-1

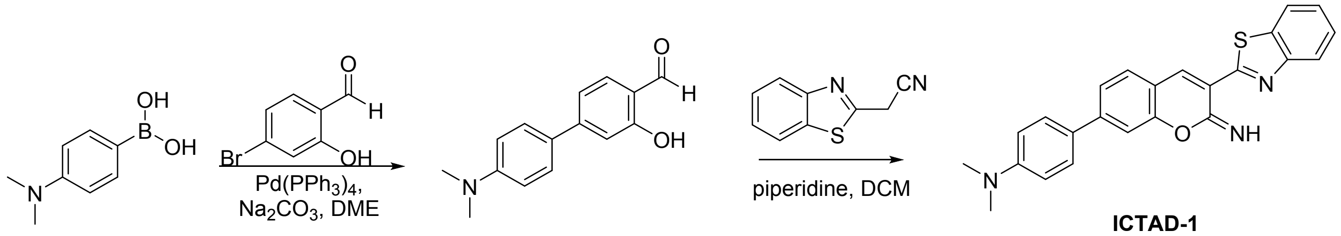

ICTAD-1

4-(Dimethylamino)phenylboronic acid (534 mg, 3.2 mmol) and 4-bromo-2-hydroxybenzaldehyde (500 mg, 2.5 mmol) were dissolved in 1,2-dimethoxyethane (35 mL). To the resulting solution, $Pd(PPh_3)_4$ (287 mg, 0.25 mmol) and a aqueous solution of sodium carbonate (2 M, 25 mL) were added under Ar_2. The mixture was heated and stirred for 5 h under 90 °C. After the reaction completed, the solution was extracted with ethyl

acetate for three times. The combined organic layers were dried by anhydrous sodium sulfate. After filtration, the solvent was removed by rotary evaporator. The residue was purified by silica gel column to give the compound 4'-(dimethylamino)-3-hydroxy-[1,1'-biphenyl]-4-carbaldehyde (422 mg, yield 70%). ^{1}H-NMR (500 MHz, $CDCl_3$) δ 11.14 (s, 1H), 9.83 (s, 1H), 7.56 (d, 2H), 7.52 (d, 1H), 7.21 (d, 1H), 7.16 (s, 1H), 6.76 (d, 2H), 3.01(s, 6H). ^{13}C-NMR (125 MHz, $CDCl_3$) δ 195.6, 162.2, 151.1, 149.9, 134.1, 128.2, 126.4, 118.7, 117.7, 113.9, 112.4, 40.4. MS: 242.1 $[M+H]^+$.

4'-(Dimethylamino)-3-hydroxy-[1,1'-biphenyl]-4-carbaldehyde (72 mg, 0.3 mmol) and 2-benzothiazoleacetonitrile (68 mg, 0.39 mmol) were dissolved in ethanol (3 mL) and dichloromethane (3 mL), followed by the addition of piperidine (1 drop). The resulting mixture was stirred for 3 h at room temperature. The formed solid was filtered and washed with ethanol to give the probe **ICTAD-1** (100 mg, Yield 84%). ^{1}H-NMR (500 MHz, $CDCl_3$) δ 8.06 (d, 1H), 7.92 (d, 1H), 7.55 (d, 2H), 7.49 (d, 2H), 7.39-7.42 (m, 3H), 7.38 (s, 2H), 6.78(d, 2H), 3.02 (s, 6H). ^{13}C-NMR (125 MHz, $CDCl_3$) δ 207.1, 154.1, 150.8, 136.1, 129.2, 127.9, 126.6, 126.4, 125.3, 122.9, 121.7, 121.5, 112.6, 112.3, 40.4. MS: 398.1 $[M+H]^+$.

Synthesis of ICTAD-2

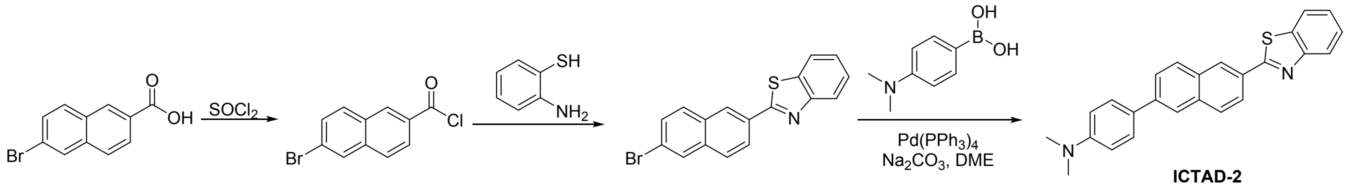

Step 1 and 2. 6-Bromo-2-naphthoic acid (1.0 g, 4.0 mmol) was added to $SOCl_2$ (10 mL), and the resulting mixture was stirred for 4 h at 80 °C. After the solvent was removed under reduced pressure, 2-aminobenzenethiol (0.6 g, 4.8 mmol) and pyridine (30 mL) were added to the residue, and the solution was stirred for 12 h at 115 °C. After the solvent was removed under reduced pressure, the resulting residue was purified by the silica gel chromatography using *n*-hexane/CH_2Cl_2 (1:1, v/v) as eluent to afford compound 2-(6-bromonaphthalen-2-yl)benzo[*d*]thiazole as a gray soild (0.98 g, 72%). ^{1}H-NMR (500 MHz, $CDCl_3$) δ 8.50 (s, 1H), 8.21 (dd, 1H), 8.10 (d, 1H), 8.02 (s, 1H), 7.91 (d, 1H), 7.82 (dd, 2H), 7.60 (dd, 1H), 7.51 (t, 1H), 7.40 (t, 1H). ^{13}C-NMR (125 MHz, $CDCl_3$) δ 167.6, 154.3, 135.6, 135.2, 131.7, 131.5, 130.4, 130.1, 127.9, 127.4, 126.6, 125.6, 125.5, 123.4, 121.8, 121.7, 31.0. MS: 340.0, 342.0 $[M+H]^+$.

Step 3. 2-(6-Bromonaphthalen-2-yl)benzo[*d*]thiazole (160 mg, 0.47 mmol) and 4-(dimethylamino)phenylboronic acid (93 mg, 0.56 mmol) were dissolved in 1,2-dimethoxyethane (10 mL). To the resulting mixture, $Pd(PPh_3)_4$ (54 mg, 0.047 mmol) and a solution of sodium carbonate (2 M, 8 mL) were added under Ar_2. The mixture was heated and stirred for 5 h under 90 °C. After the reaction completed, the solution was extracted with ethyl acetate for three times. The combined organic layers were dried by anhydrous sodium sulfate. After filtration, the solvent was removed by rotary evaporator, and the resulting residue was purified by silica gel column to give **ICTAD-2** (139 mg, yield 78%). ^{1}H-NMR (500 MHz, $CDCl_3$) δ 8.54 (s, 1H), 8.18 (d, 1H), 8.10 (d, 1H), 8.00 (s, 1H), 7.96-7.99 (m, 3H), 7.93 (d, 1H), 7.65 (d, 2H), 7.50 (t, 1H), 7.39 (t, 1H), 6.84 (d, 2H), 3.02 (s, 6H). ^{13}C-NMR (125 MHz, $CDCl_3$) δ 207.1, 168.4, 154.4, 150.3, 140.3, 135.3, 135.2, 131.8, 130.4, 129.2, 128.9, 128.3, 128.1, 127.4, 126.4, 126.3, 125.2, 124.8, 124.0, 123.2, 121.7,

112.9, 40.6. MS: 381.1 [M+H]+.

Synthesis of ICTAD-3

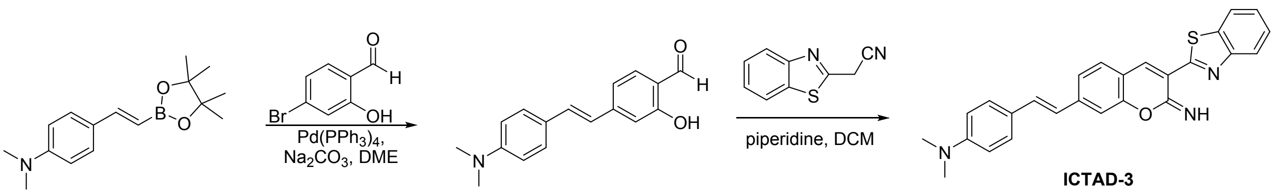

ICTAD-3

Step 1. 2-(4-*N*,*N*-dimethylphenyl)vinylboronic acid pinacol ester (328 mg, 1.2 mmol) and 4-bromo-2-hydroxybenzaldehyde (201 mg, 1.0 mmol) were dissolved in 1,2-dimethoxyethane (20 mL). To the resulting mixture, $Pd(PPh_3)_4$ (115 mg, 0.1 mmol) and a solution of sodium carbonate (2 M, 10 mL) were added under Ar_2. The mixture was heated and stirred for 7 h under 90 °C. After the reaction completed, the solution was extracted with ethyl acetate for three times. The combined organic layers were dried by anhydrous sodium sulfate. After filtration, the solvent was removed by rotary evaporator to provide a semi-solid residue. To the resulting residue, a mixture of ethyl acetate and n-hexane (1:1, v/v, 10 mL) was added and white precipate was formed. The precipate was filtered to afford (*E*)-4-(4-(dimethylamino)styryl)-2-hydroxybenzaldehyde, which was used for next step without further purification.

Step 2. (*E*)-4-(4-(dimethylamino)styryl)-2-hydroxybenzaldehyde (75 mg, 0.27 mmol) and 2-benzothiazoleacetonitrile (49 mg, 0.28 mmol) were dissolved in ethanol (10 mL) and dichloromethane (2 mL), followed by the addition of piperidine (1 drop). The resulting solution was stirred for 3 h at room temperature. The formed solid was filtered and washed with ethanol to give the compound **ICTAD-3** (60 mg, Yield 52%). ^{1}H-NMR (500 MHz, $CDCl_3$) δ 8.05 (d, 1H), 7.92 (d, 1H), 7.49-7.41 (m, 6H), 7.28-7.38 (m, 1H), 7.27 (s, 1H), 7.22 (d, 1H), 6.86 (d, 1H), 6.69 (d, 2H), 2.99 (s, 6H). ^{13}C-NMR (125 MHz, $CDCl_3$) δ 207.1, 154.0, 152.7, 150.7, 143.1, 135.9, 132.3, 129.0, 128.3, 126.3, 125.3, 124.7, 122.9, 121.9, 121.5, 117.6, 112.3, 112.2, 40.4 MS: 424.2 [M+H]+.

Synthesis of ICTAD-4

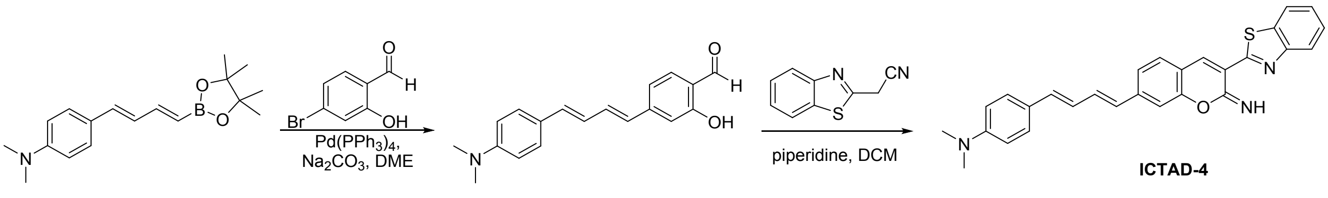

ICTAD-4

ICTAD-4 was obtained by following the similar procedure as ICTAD-3. ^{1}H-NMR (500 MHz, $CDCl_3$) δ 8.05 (d, 1H), 7.91 (d, 1H), 7.48-7.50 (m, 1H), 7.33-7.39 (m, 5H), 7.21 (d, 1H), 7.16 (s, 1H), 7.03-7.05 (m, 1H), 6.76-6.79 (m, 1H), 6.66-6.74 (m, 3H), 6.52 (d, 1H), 2.98 (s, 6H). ^{13}C-NMR (125 MHz, $CDCl_3$) δ 207.1, 153.9, 150.5, 135.9, 133.5, 129.0, 128.5, 128.0, 126.4, 125.3, 124.4, 123.0, 122.0, 121.5, 112.3, 40.4. MS: 450.1 [M+H]+.

Table S1. The summary of properties of ICTAD-1 in different organic solvents.

solvent	polarity	QY	Max emission wavelength (nm)
Hexane	0.06	1.26	514
Toluene	2.4	0.84	537
Ethyl ether	2.9	0.75	550
Chloroform	4.4	0.58	570
EA	4.3	0.57	581
THF	4.2	0.56	588
Isopropanol	4.3	0.16	625
Acetone	5.4	0.21	630
DMF	6.4	0.11	644
Acetonitrile	6.2	0.08	646
Methanol	6.6	0.01	650
DMSO	7.2	0.06	658
Ethylene glycol	6.9	0.02	660

QY: quantum yield, determined versus Rhodamine B in ethanol (0.65)

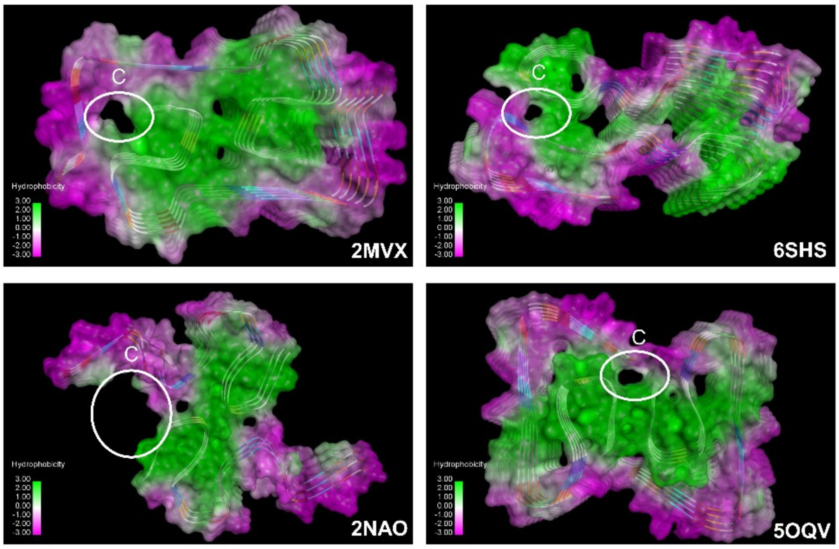

Figure S1. Binding sites C of Aβ proteins. (Aβ 40: 2MVX and 6SHS; Aβ 42: 2NAO and 5OQV).

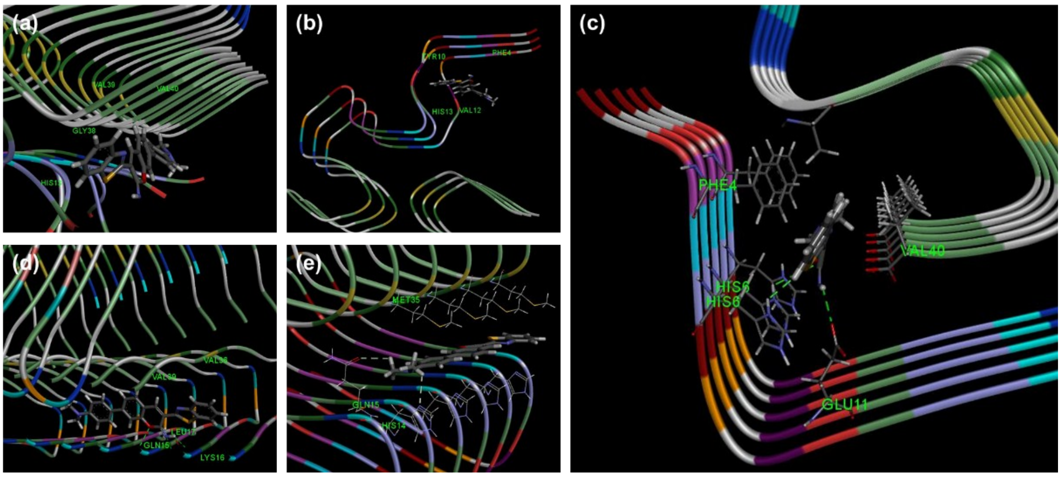

Figure S2. The binding models of probe ICTAD-1 with Aβ fibrils (a) PDB: 5KK3; (b) PDB: 2NAO; (c) PDB: 2MVX; (d) PDB: 2MMPZ; (e) PDB: 6SHS.

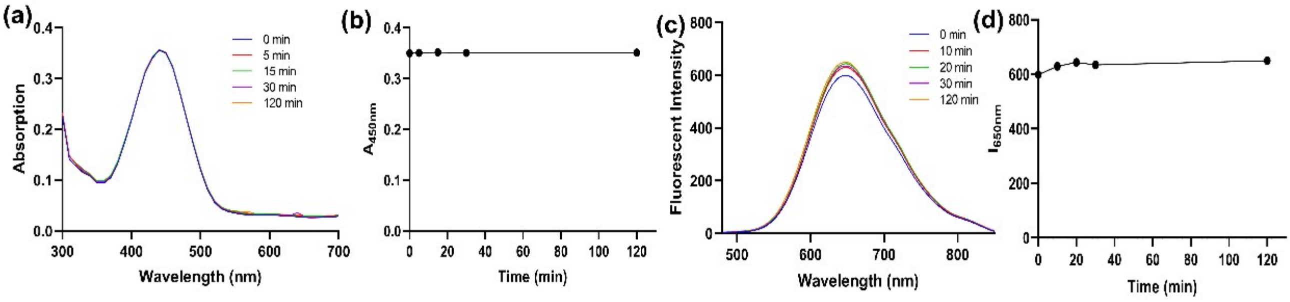

Figure S3. Light stability of ICTAD-1. The absorption (a) and fluorescent spectra (b) changes of ICTAD-1 (10 μM) in DMF under the irradiation of daylight lamp for 120 min; the quantification of absorbance at 450 nm (a) or fluorescent intensity at 650 nm (b) of ICTAD-1 (10 μM) in DMF under the irradiation of daylight lamp within 120 min.

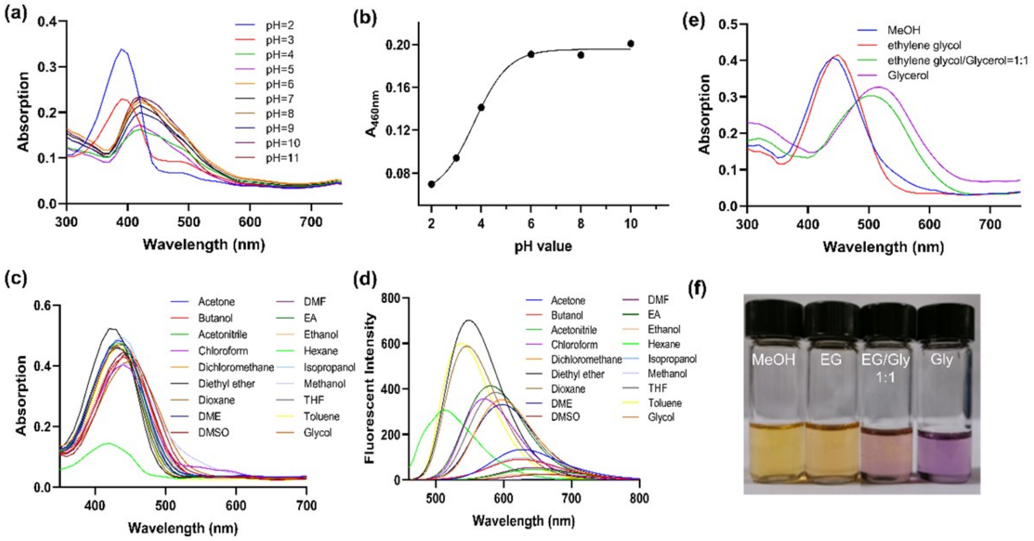

Figure S4. (a) The absorption spectra of ICTAD-1 (10 μM) in different pH aqueous solutions; (b) the fitted curve between pH values and absorbance at 460 nm; (c) the absorption spectra of ICTAD-1 (10 μM) in different solvents; (d) the fluorescent spectra of ICTAD-1 (10 μM) in different solvents; (e) the absorption spectra of ICTAD-1 (10 μM) in solvent with different viscosity; (f) the picture of ICTAD-1 (10 μM) in solvent with different viscosity.

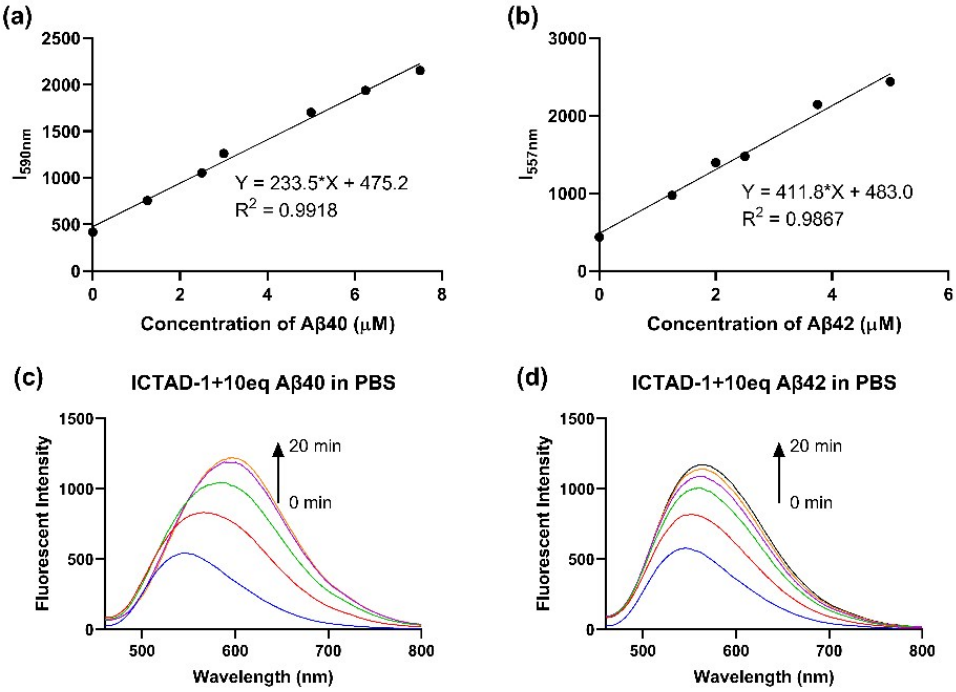

Figure S5. (a) the linear relationship between the concentration of Aβ40 (0-7.5 μM) and the fluorescent intensities at 590 nm; (b) the linear relationship between the concentration of Aβ42 (0-5 μM) and the fluorescent intensities at 557 nm; (c-d)Time dependence of ICTAD-1 (250 nM) toward Aβ (10 eq, 2.5 μM) in PBS (10 mM, pH7.4, 1% DMF).

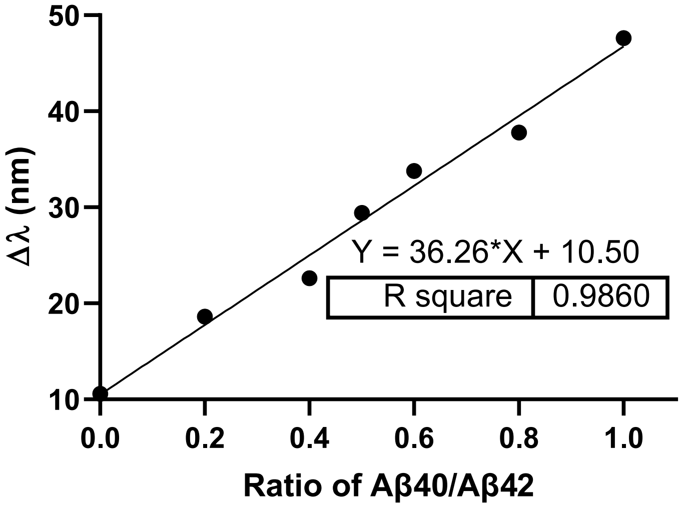

Figure S6. The linear relationship between the ratio of Aβ40/Aβ42 and the redshifted wavelength.

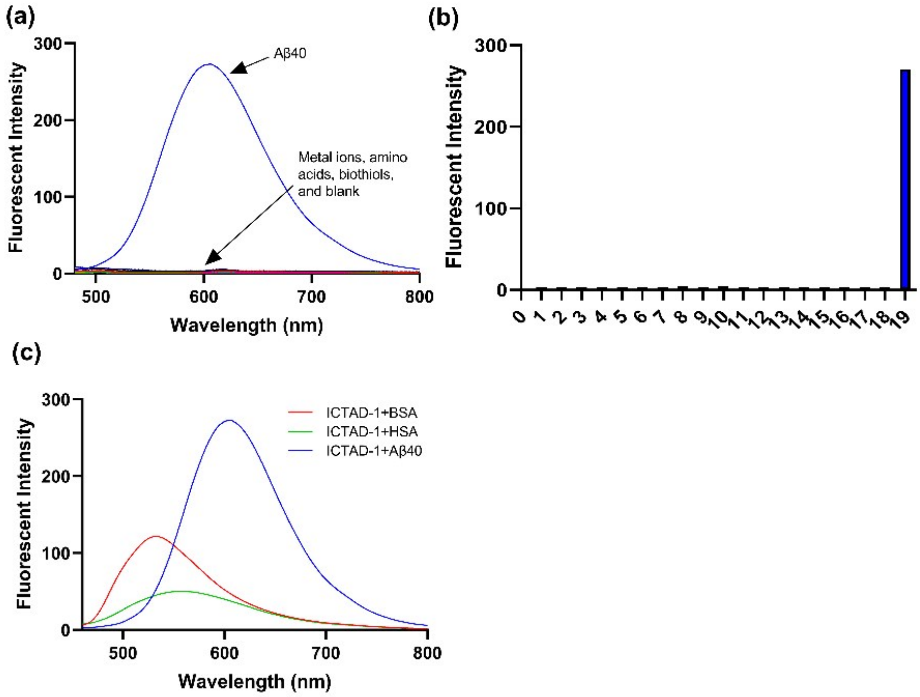

Figure S7. (a) Selectivity of ICTAD-1 (10 μM) toward Aβ. (1. Blank; 2. Na^+; 3. K^+; 4. Ca^{2+}; 5. Mg^{2+}; 6. Zn^{2+}; 7. Cu^{2+}; 8. Mn^{2+}; 9. Al^{3+}; 10. Fe^{3+}; 11. Arg; 12. Asp; 13. Glu; 14. His; 15. Leu; 16. Lys; 17. Cys; 18. GSH; 19. Aβ); (b) The qualification of fluorescent intensities; (c) The fluorescence spectra of ICTAD-1 (10 μM) in the presence of HSA (10 μM) or BSA(10 μM).

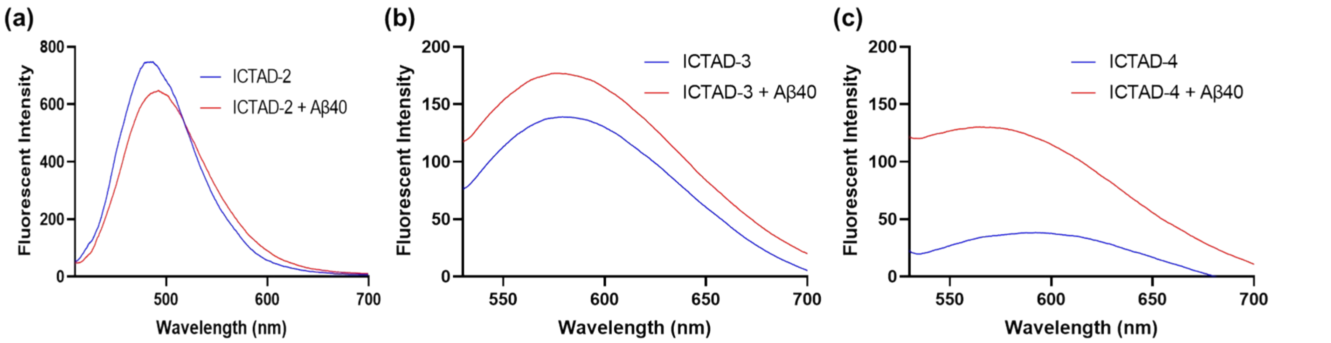

Figure S8. Fluorescent spectra of ICTAD-2, ICTAD-3, and ICTAD-4 (250nM) in the absence or presence of Aβ40 (250 nM) in PBS (10 mM, pH 7.4, 1% DMF)

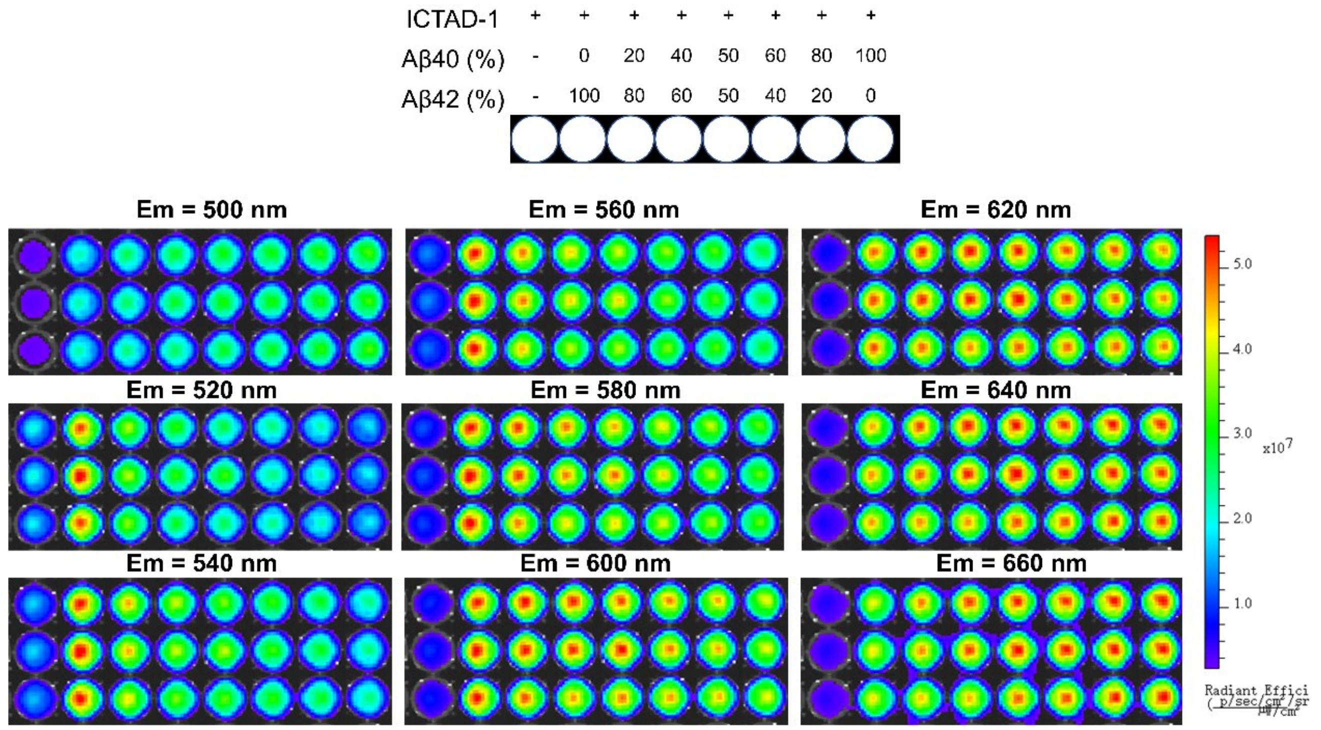

Figure S9. Raw images of plate imaging with ICTAD-1 (250 nM) in the presence of different ratios of Aβ40/Aβ42 (2.5 μM, 10 eq) in 96-wells plate before unmixing.

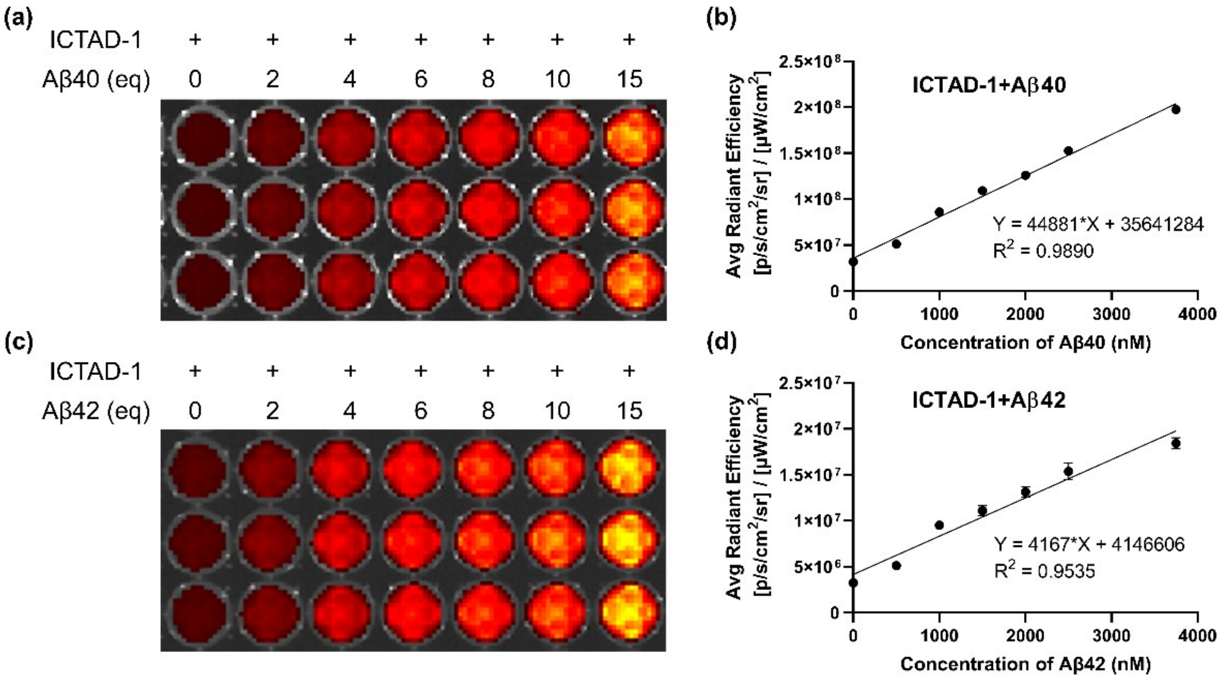

Figure S10. (a) Plate imaging of ICTAD-1 (250 nM) in the presence of different concentrations of Aβ40 (0- 4 μM) in 96-wells plate. E_x= 430 nm, E_m = 620 nm. (b) the linear relationship between the concentration of Aβ40 and the average radiant efficiency. (c) Plate imaging of ICTAD-1 (250 nM) in the presence of different concentrations of Aβ42 (0- 4 μM) in 96-wells plate. E_x= 430 nm, E_m = 580 nm. (d) the linear relationship between the concentration of Aβ42 and the average radiant efficiency.

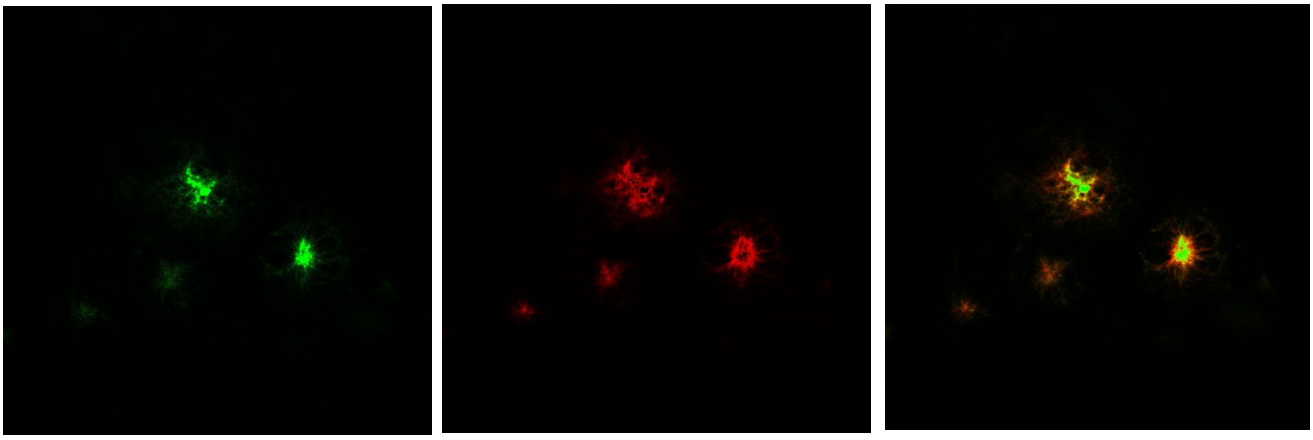

Figure S11. Confocal spectral unmixing for multiple plaques to dissect the signals from Aβ42 (green) and Aβ40 (red).

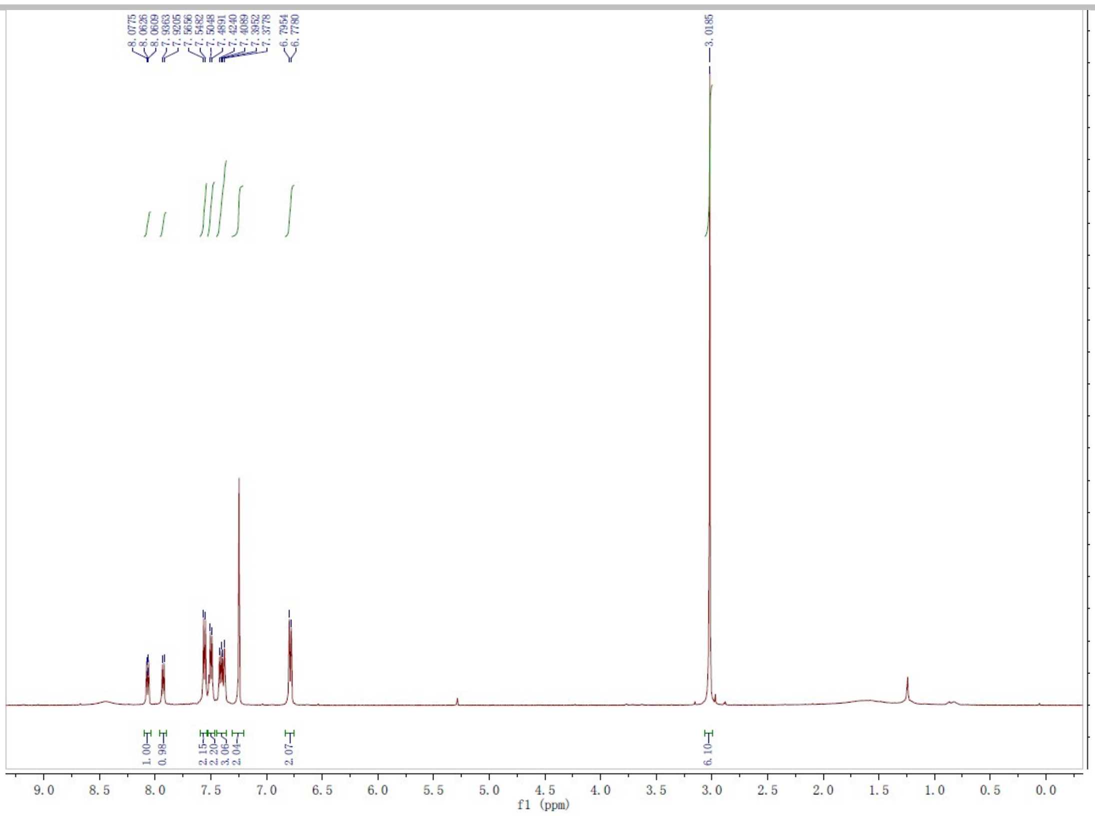

Figure S12. ^{1}H-NMR spectrum of ICTAD-1

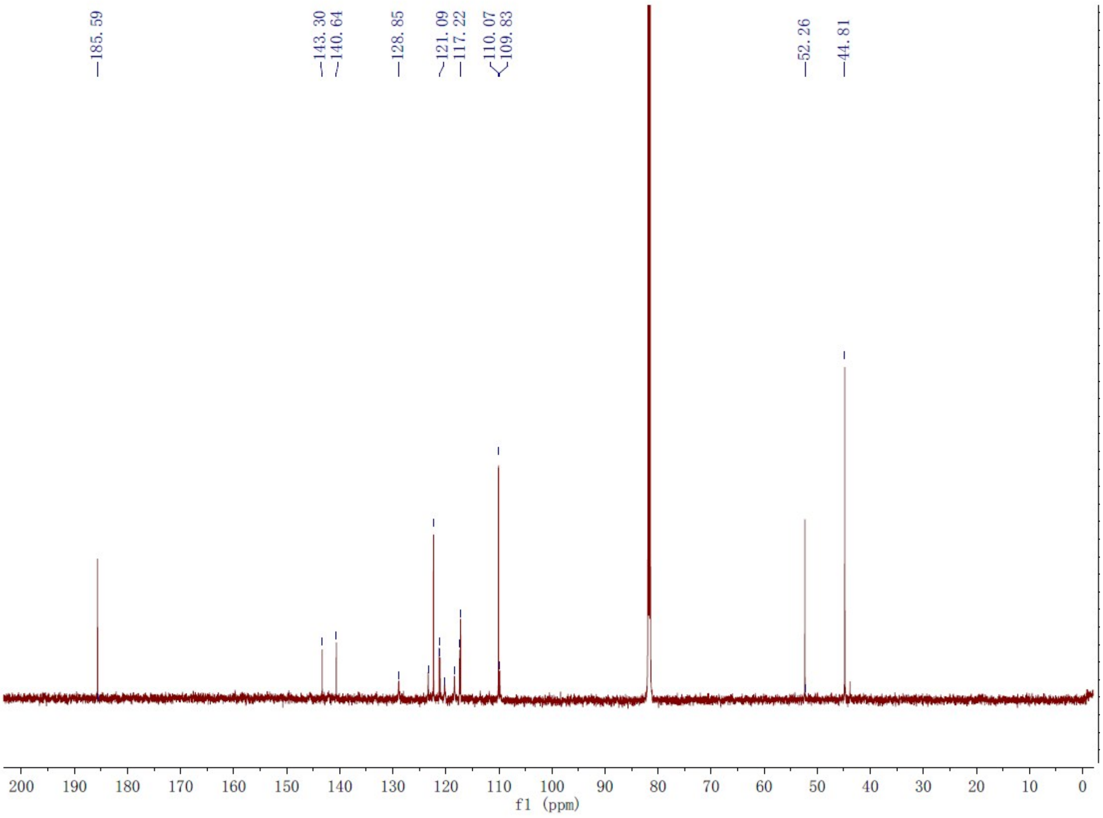

Figure S13. ^{13}C-NMR spectrum of ICTAD-1

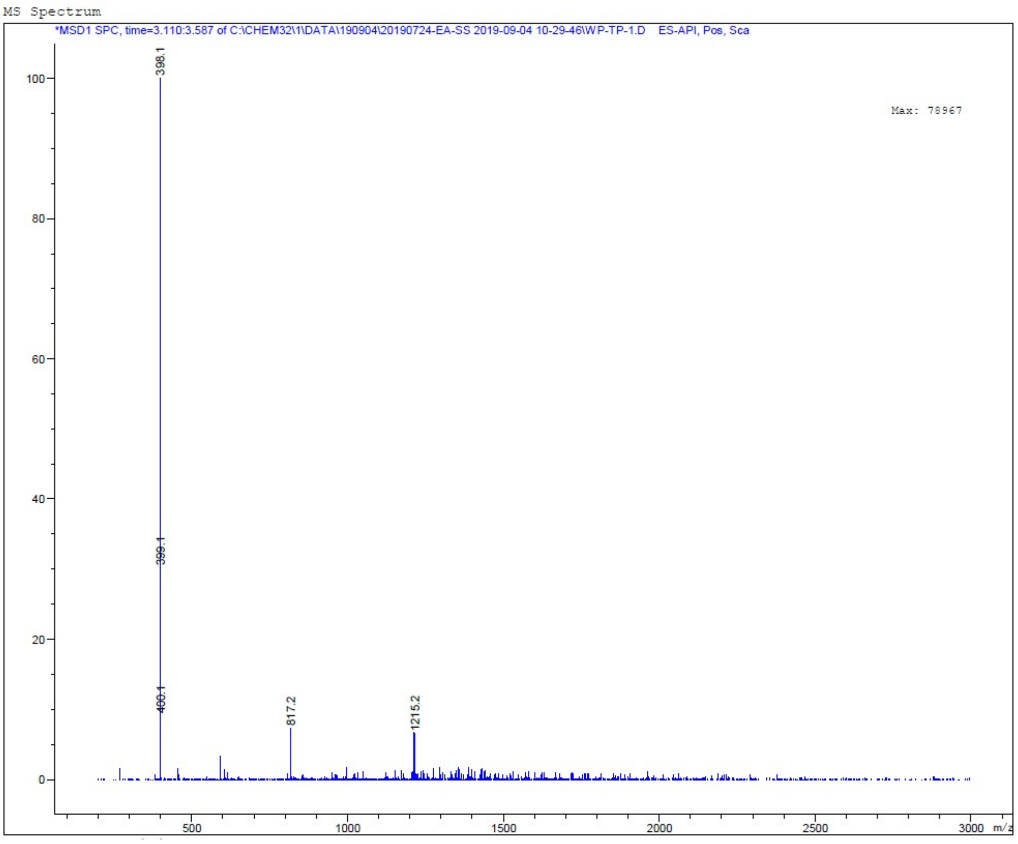

Figure S14. MS spectrum of ICTAD-1

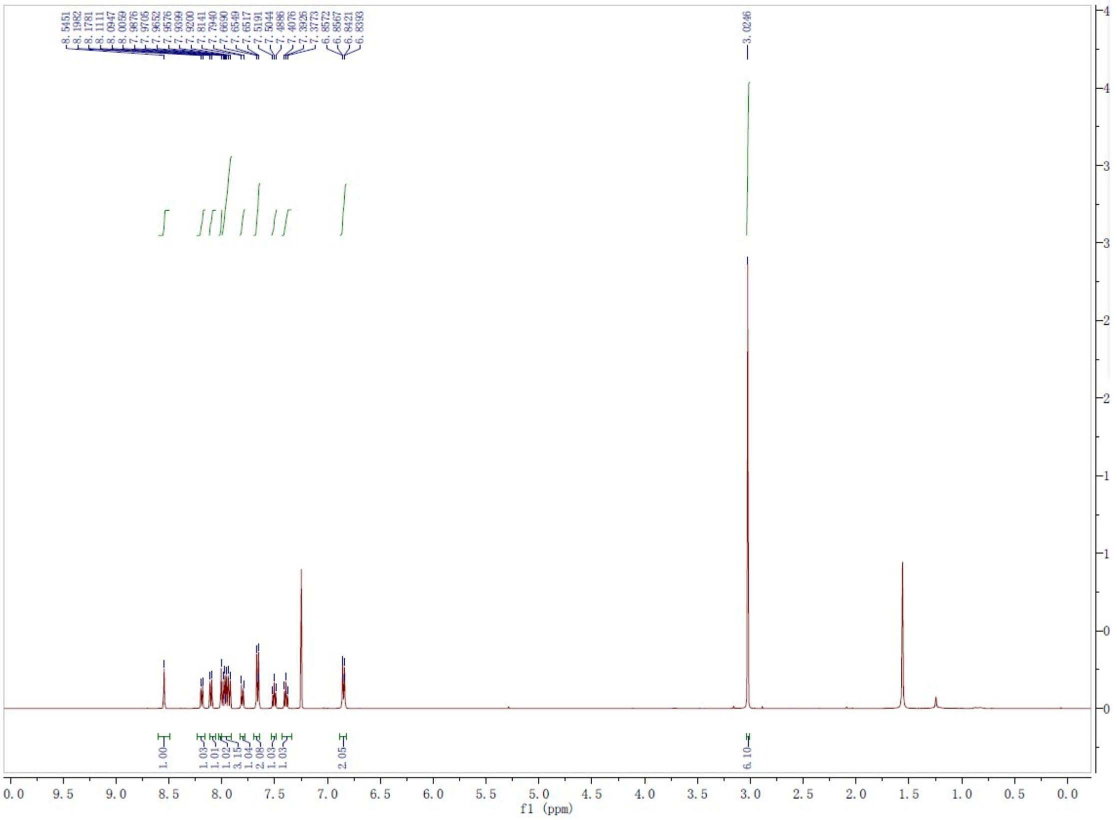

Figure S15. ^{1}H-NMR spectrum of ICTAD-2

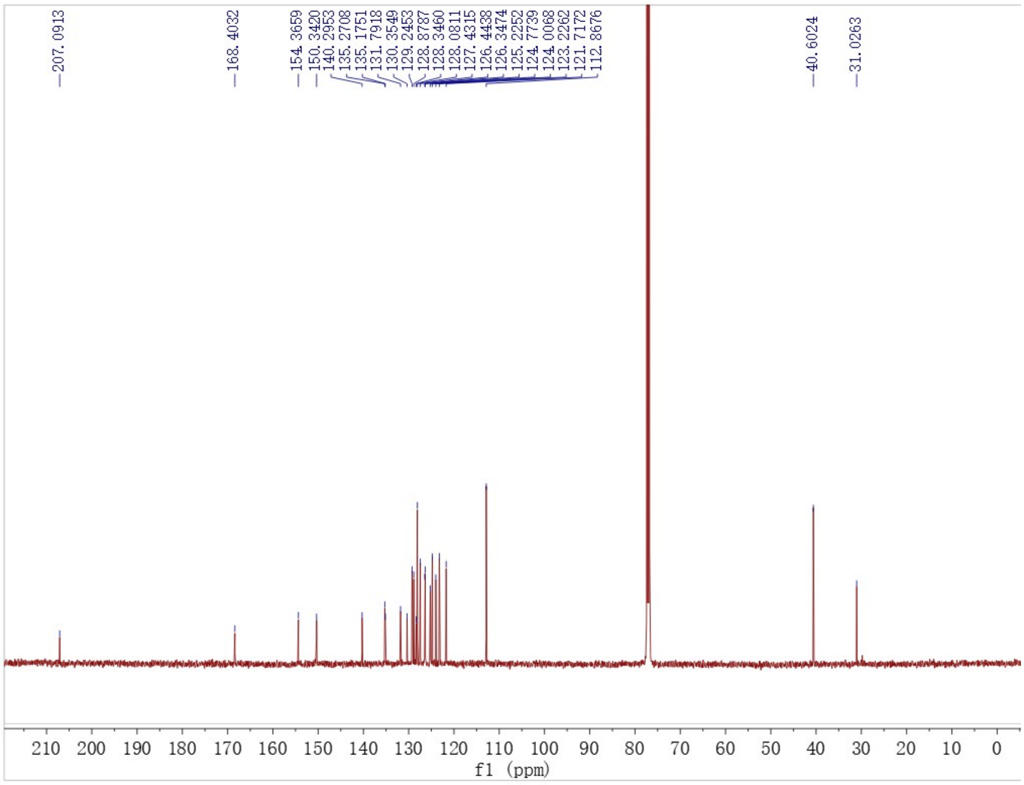

Figure S16.^{13}C-NMR spectrum of ICTAD-2

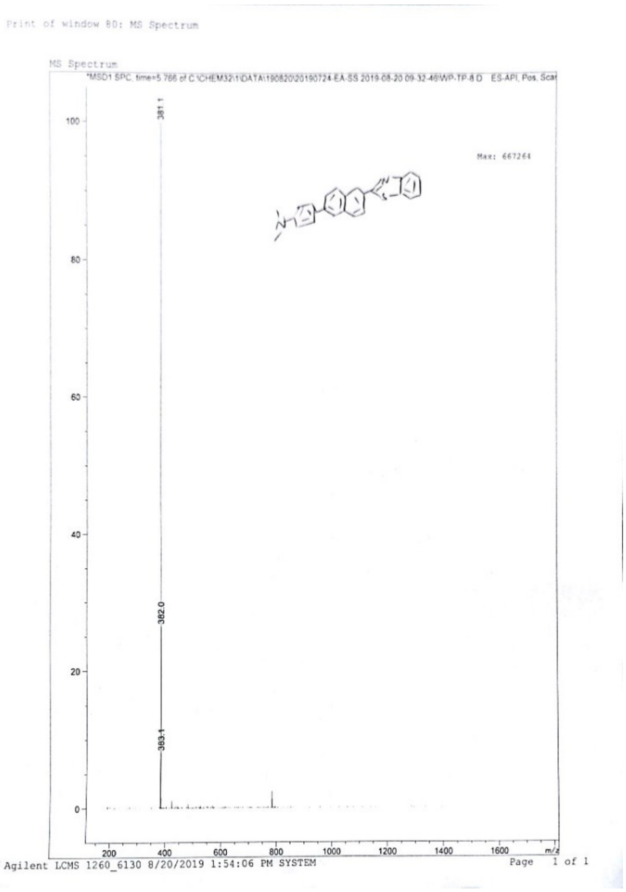

Figure S17. MS spectrum of ICTAD-2

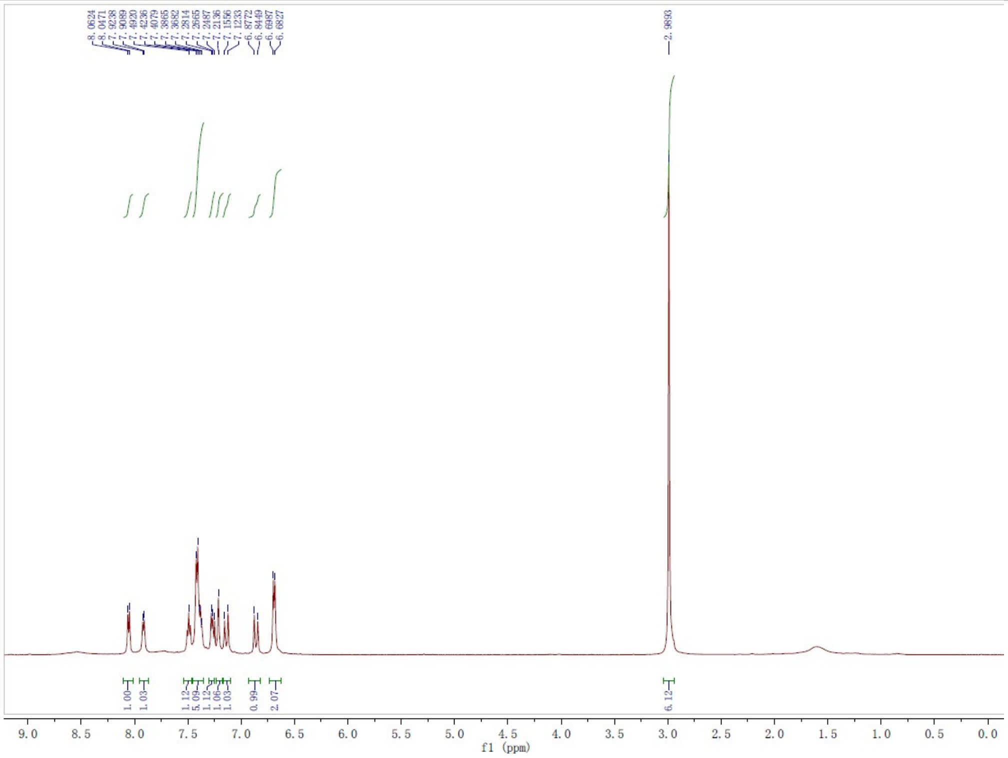

Figure S18. ^{1}H-NMR spectrum of ICTAD-3

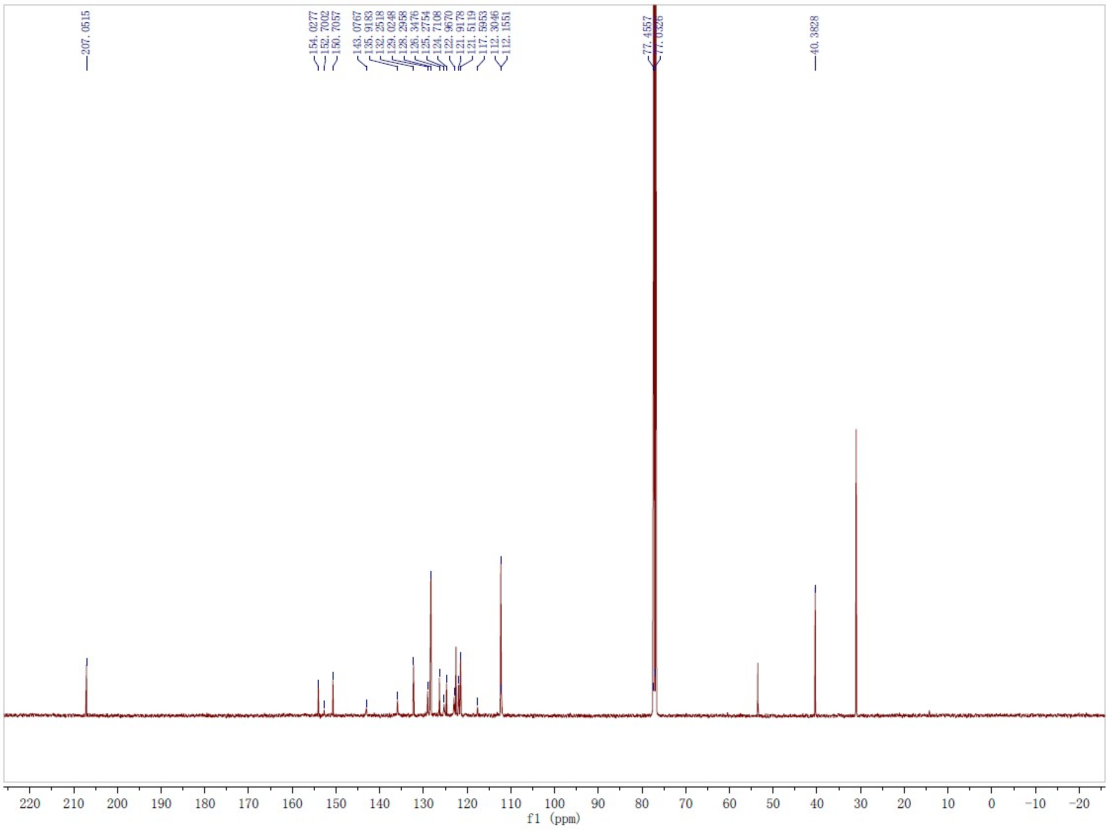

Figure S19. ^{13}C-NMR spectrum of ICTAD-3

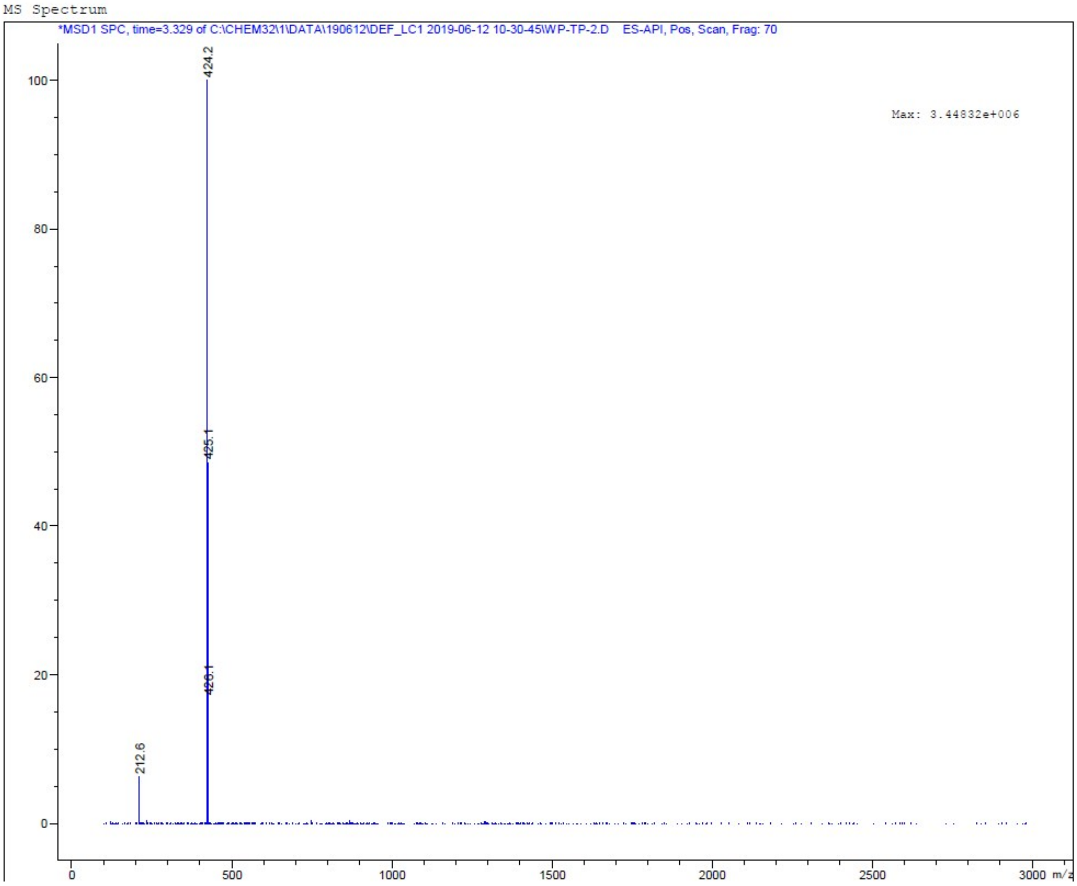

Figure S20. MS spectrum of ICTAD-3

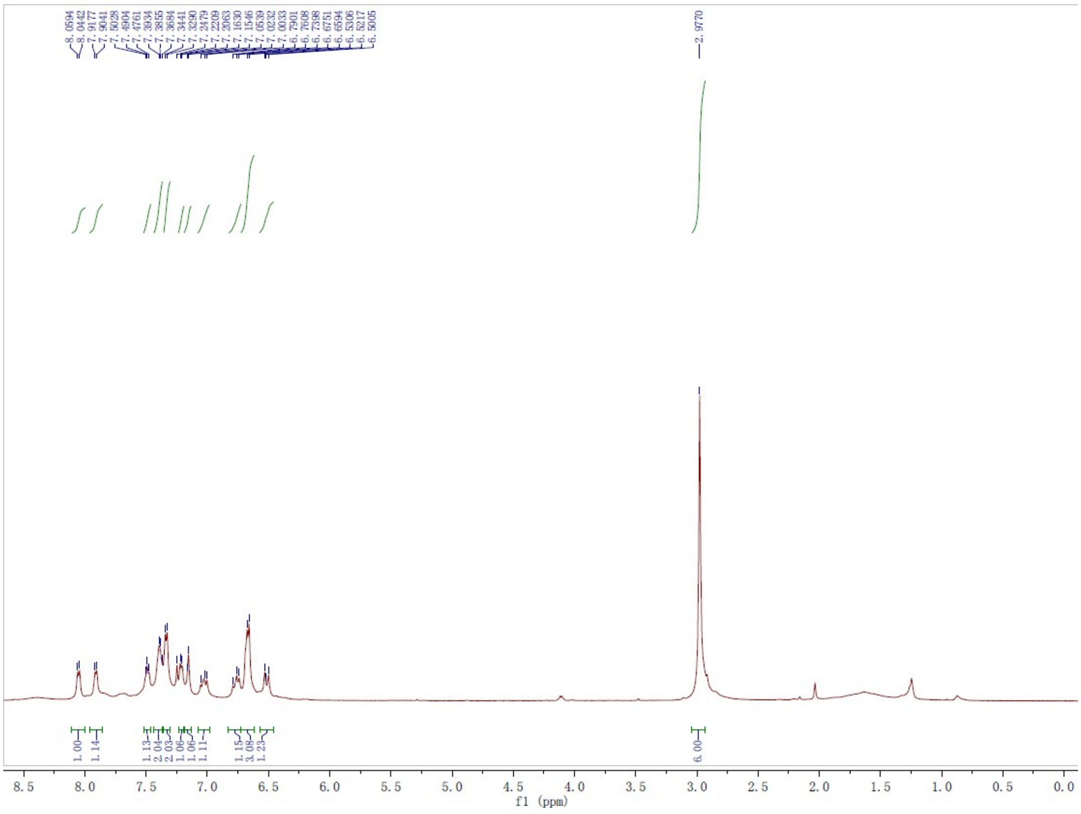

Figure S21. ^{1}H-NMR spectrum of ICTAD-4

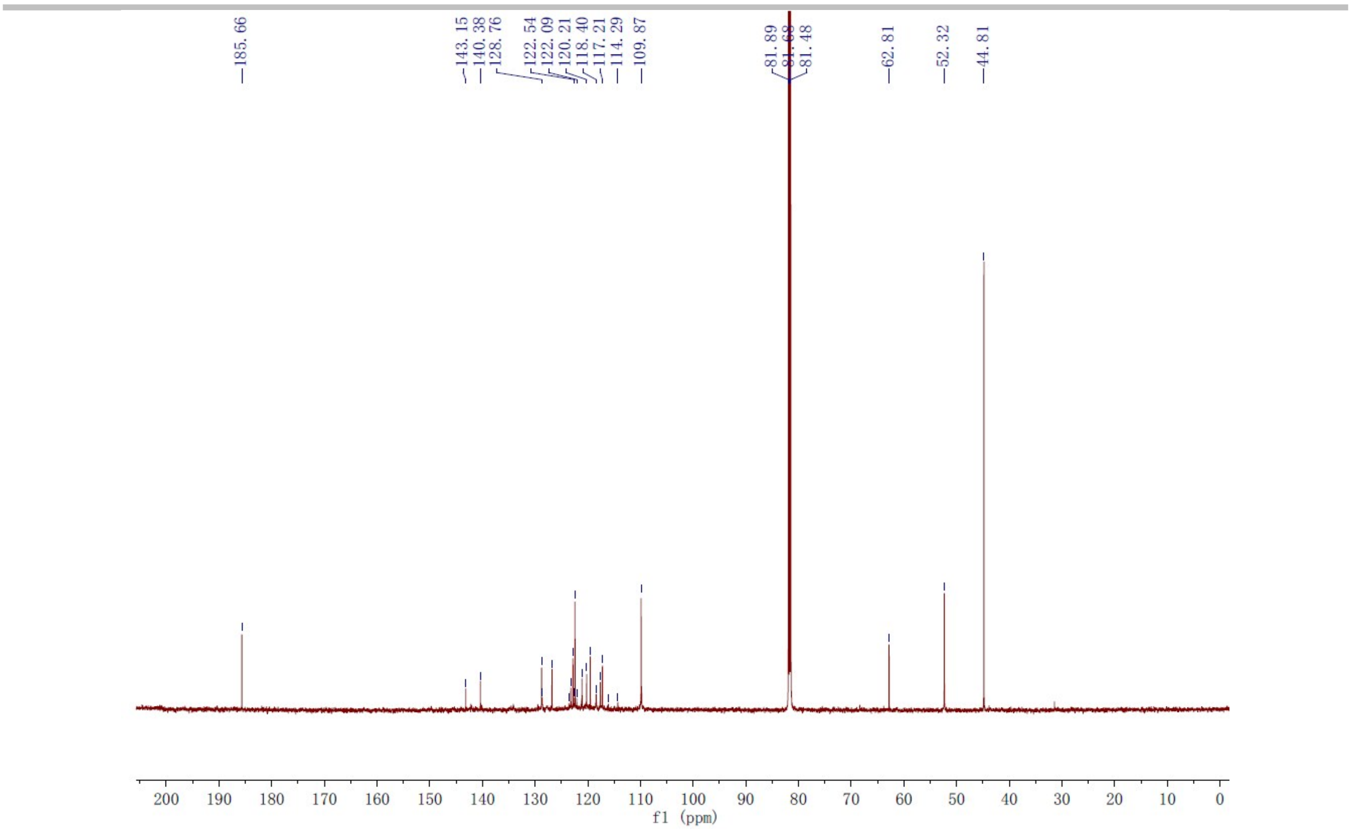

Figure S22. ^{13}C-NMR spectrum of ICTAD-4

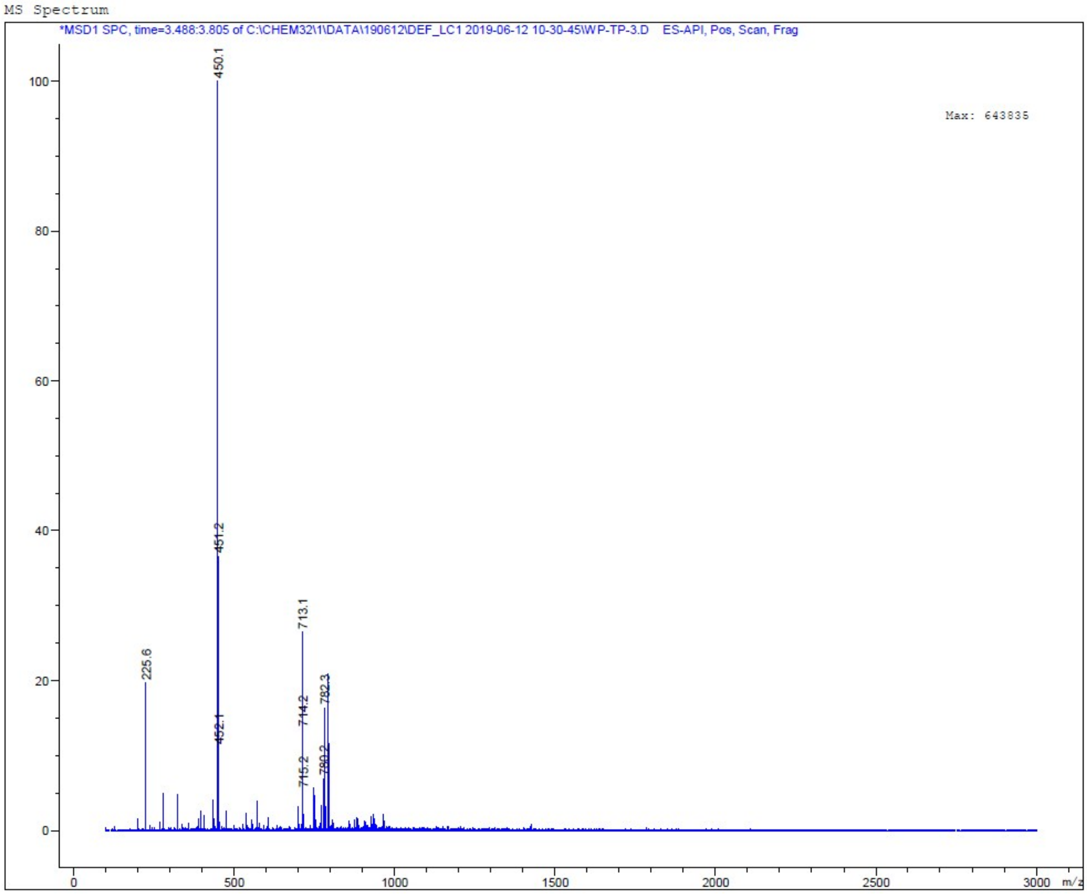

Figure S23. MS spectrum of ICTAD-4
